# Selective reaction monitoring approach using structure-defined synthetic glycopeptides for validating glycopeptide biomarkers pre-determined by bottom-up glycoproteomics†

**DOI:** 10.1039/d2ra02903k

**Published:** 2022-08-03

**Authors:** Kouta Shiratori, Yasuhiro Yokoi, Hajime Wakui, Nozomi Hirane, Michiru Otaki, Hiroshi Hinou, Tohru Yoneyama, Shingo Hatakeyama, Satoshi Kimura, Chikara Ohyama, Shin-Ichiro Nishimura

**Affiliations:** Field of Drug Discovery Research, Faculty of Advanced Life Science, and Graduate School of Life Science, Hokkaido University N21 W11, Kita-ku Sapporo 001-0021 Japan shin@sci.hokudai.ac.jp; ENU Pharma, Co., Ltd N7, W6, Kita-ku Sapporo 060-0807 Japan; Department of Urology, Graduate School of Medicine, Hirosaki University Hirosaki 036-8562 Japan; Department of Laboratory Medicine and Central Clinical Laboratory, Showa University, Northern Yokohama Hospital Yokohama 224-8503 Japan

## Abstract

Clusterin is a heavily glycosylated protein that is upregulated in various cancer and neurological diseases. The findings by the Hancock and Iliopoulos group that levels of the tryptic glycopeptide derived from plasma clusterin, ^372^Leu-Ala-Asn-Leu-Thr-Gln-Gly-Glu-Asp-Gln-Tyr-Tyr-Leu-Arg^385^ with a biantennary disialyl *N*-glycan (A2G2S2 or FA2G2S2) at Asn374 differed significantly prior to and after curative nephrectomy for clear cell renal cell carcinoma (RCC) patients motivated us to verify the feasibility of this glycopeptide as a novel biomarker of RCC. To determine the precise *N*-glycan structure attached to Asn374, whether A2G2S2 is composed of the Neu5Acα2,3Gal or/and the Neu5Acα2,6Gal moiety, we synthesized key glycopeptides having one of the two putative isomers. Selective reaction monitoring assay using synthetic glycopeptides as calibration standards allowed “top-down glycopeptidomics” for the absolute quantitation of targeted label-free glycopeptides in a range from 313.3 to 697.5 nM in the complex tryptic digests derived from serum samples of RCC patients and healthy controls. Our results provided evidence that the Asn374 residue of human clusterin is modified dominantly with the Neu5Acα2,6Gal structure and the levels of clusterin bearing an A2G2S2 with homo Neu5Acα2,6Gal terminals at Asn374 decrease significantly in RCC patients as compared with healthy controls. The present study elicits that a new strategy integrating the bottom-up glycoproteomics with top-down glycopeptidomics using structure-defined synthetic glycopeptides enables the confident identification and quantitation of the glycopeptide targets pre-determined by the existing methods for intact glycopeptide profiling.

## Abstract 2

Structure-defined synthetic glycopeptides allow the validation of glycopeptide biomarkers pre-determined from bottom-up glycoproteomics based on the selective reaction monitoring approach.

## Graphical abstract

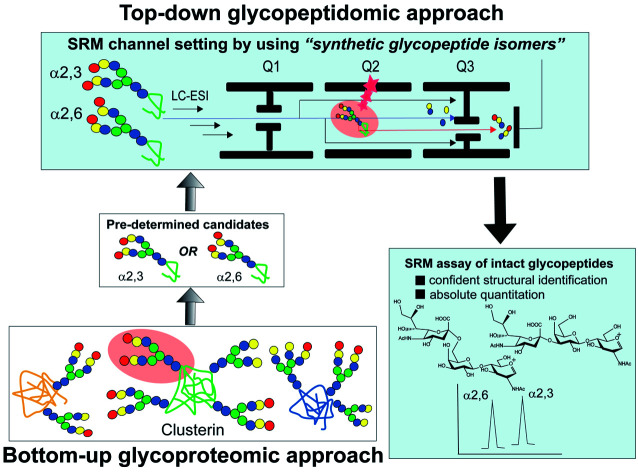

## Introduction

Post-translational protein glycosylation influences a diverse number of crucial biological processes, including cellular differentiation, recognition, adhesion, and maintenance/regulation of the homeostatic immune responses.^1^ It is clear that glycosylation alterations are involved in several key steps of cancer pathogenesis such as tumour growth, progression, and metastasis.^2^ Accumulating results provide evidence that specific *N*- or *O*-glycosylation of cell surface proteins is crucial for cancer cells that escape immune surveillance through the interaction of these glycoprotein ligands with some immune cell receptors.^3^ Recent studies have demonstrated that site-specific *N*-glycosylation of programmed cell death ligand-1 (PD-L1) at Asn192, Asn200, and Asn219 is essential for the direct binding of PD-L1 with the T-cell receptor programmed cell death protein-1 (PD-1), a key step for the PD-L1-mediated immunosuppressive functions acquired by many tumour cells.^4,5^ It is also interesting to note that multiple *O*-glycosylations of mucin glycoprotein MUC1 tandem repeats with sialyl T-antigen drives the differentiation of human monocytes into tumour-associated macrophages through engagement with sialic acid-binding immunoglobulin-like lectin 9 (Siglec-9).^6,7^ Interactions between various Gal-terminated *N*-/*O*-glycans of cancer-related proteins and galectins are also known to contribute to the immunosuppressive potential of tumour cells by impairing T cell functions, inducing the differentiation of tolerogenic dendritic cells (DCs) and tumour-associated macrophage, and modulating natural killer (NK) cell activity in a variety of tumors.^8,9^

Despite the emerging importance of dynamic protein glycosylation in cancer and many other diseases, the molecular basis for changes in the degree of glycan occupations and their structures (glycoforms) at the potential glycosylation sites of proteins, leading to the range of clinical symptoms associated with the diseases, remains largely unknown.^2,3^ Our interest has been focused on the significance of site-specific glycosylation of cancer-related proteins in the creation of tumour-associated glycopeptidic neoepitopes, namely “dynamic epitopes” as novel diagnostic biomarkers and molecular targets for the development of anticancer therapeutic monoclonal antibodies (mAbs).^10–12^ Intriguingly, binding specificity and affinity of galectins,^13^ macrophage galactose-binding lectin (MGL),^14^ as well as siglecs^6,7^ with carbohydrate ligands, can be affected significantly by three-dimensional structures of the proximal peptide region including the glycosylated site, indicating that tissue-resident lectins may recognize the dynamic epitopes by concurrently interacting with both the carbohydrate part and the attached core peptide moiety.

Clusterin (Apolipoprotein J), a heavily glycosylated protein that harbours seven potential *N*-glycosylation sites, is highly expressed in the liver, brain, and kidney and is found in blood plasma.^15^ Clusterin has been shown to be involved in carcinogenesis-related cell events such as DNA repair, apoptosis, adhesion, and tissue remodeling.^16^ The significance of clusterin differential expression has been reported in various cancers such as breast, prostate, ovarian, and RCC.^17–21^ Notably, changes in the expression levels of secretory clusterin have been found in different types of RCC cell lines and tissues, in which clusterin has also been shown to contribute to the anti-tumour activity of the von Hippel-Linden (VHL) protein.^18,19^ Therefore, the secreted form of clusterin in RCC patients was expected to be a potent predictor of disease extension. However, in clear cell RCC (ccRCC) patients with VHL disease, it was also indicated that the loss of the VHL tumour suppressor gene has been associated with a hypoxia-inducible factor (HIF)-independent upregulation of clusterin in tumour cells.^22^ These results imply that strong expression of clusterin in surgically removed ccRCC tissue may correlate with anti-apoptotic and shorter recurrence-free survival of RCC patients with VHL disease.^23^ Given the pathological complexity of RCC and unclear biological role of clusterin, it seems likely that changes in the serum levels of circulating clusterin do not simply serve as an ideal biomarker for RCC detection and for monitoring disease progression.

**Fig. 1:**
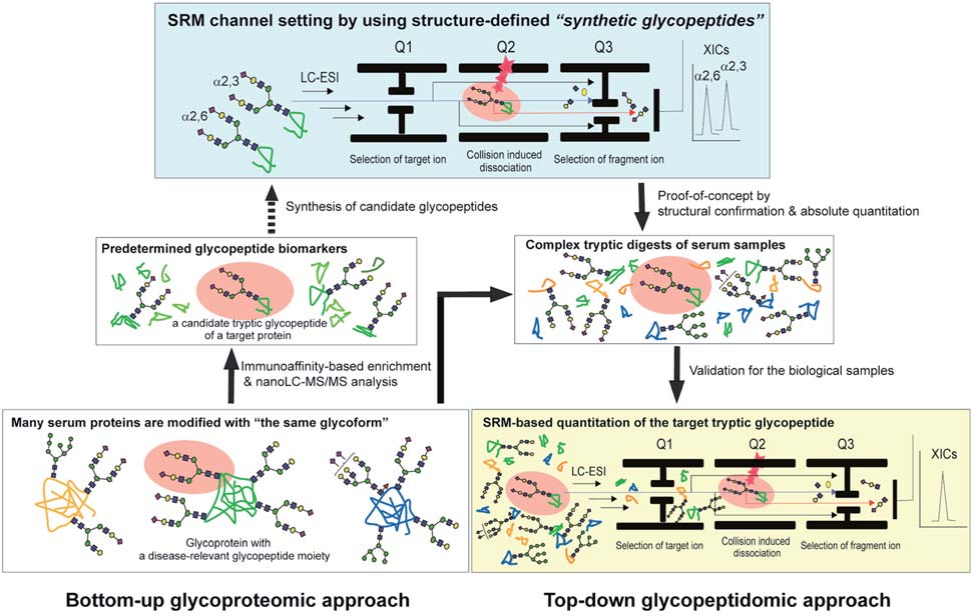
The glycoprotein-focused bottom-up glycoproteomics to top-down glycopeptidomic approach using “synthetic glycopeptides”. Glycopeptidic biomarkers pre-determined from purified human clusterin by the Iliopoulos group^24^ were synthesized and employed for calibration standards in the SRM-based absolute quantitation of the targeted glycopeptides generated by the tryptic digestion of whole serum glycoproteins, namely the top-down glycopeptidomic approach. A specific fragment ion generated from the target analyte (selected glycopeptide, Q1) under the optimized collision energy (Q2) is selected in Q3 and guided to the detector. The number of target fragment ions such as mono- and oligosaccharide oxonium ions is counted over time, resulting in an SRM trace for each target glycopeptide. Notably, the SRM assay facilitates the accurate quantitation of the targeted glycopeptide without the influence of the large excess of other tryptic digests by using the designated SRM channel (Q1, Q2, and Q3, as well as LC retention time) optimized for each targeted glycopeptide. LC-ESI, liquid chromatography-electrospray ionization; XICs, extracted ion chromatograms.

Pioneering work by the Hancock and Iliopoulos group showed the importance of site-specific clusterin glycosylation at the Asn374 residue as distinctively potential biomarkers for ccRCC when the glycan occupancy and the degree of glycoform heterogeneity were compared with those of six other potential glycosylation sites, Asn86, Asn103, Asn145, Asn291, Asn317, and Asn354.^24^ They demonstrated by a bottom-up glycoproteomic approach using nano-LC-MS/MS and lectin blotting that levels of the tryptic glycopeptides derived from immunoaffinity-purified plasma clusterin, ^372^Leu-Ala-Asn-Leu-Thr-Gln-Gly-Glu-Asp-Gln-Tyr-Tyr-Leu-Arg^385^ having a biantennary disialyl *N*-glycan (A2G2S2 or FA2G2S2) at Asn374, differed significantly prior to and after curative nephrectomy for ccRCC patients. We hypothesized that the selective reaction monitoring (SRM) assay of the glycopeptides^25–27^ by means of the structure-defined synthetic glycopeptides as calibration standards may directly enable the absolute quantitation of the intact clusterin glycopeptides carrying a precise glycoform at the Asn374 residue even in the presence of a large excess of other peptide/glycopeptide fragments produced by tryptic digestion of the complex serum samples (Fig. 1). Notably, characteristic sugar oxonium ions derived specifically from glycopeptides that can be used as transitions in SRM mode facilitate the accurate quantitation of label-free glycopeptides without multiple processes to optimize transitions for each specific glycopeptide. Merits of the SRM-based approach have been reported by the quantitative analysis of Herceptin-derived IgG1 Fc fragments having a scarce bisect-type *N*-glycoform^28^ and the serum α1-acid glycoprotein-derived fragment bearing a triantennary *N*-glycan,^29^ in which the rapid and efficient synthesis of the pre-determined complex glycopeptides is critical for achieving the desired SRM measurements.^30–34^ Importantly, our approach will allow for the discrimination and quantitation of regiospecific isomers (glycopeptide variants) that can be produced biosynthetically by the distal sialylation, whether Neu5Acα2,3Gal or/and Neu5Acα2,6Gal structure(s) in the glycosylation at Asn374. In the present study, we show that serum clusterin is modified at the Asn374 residue dominantly with a homo sialyl A2G2S2 *N*-glycan with two Neu5Acα2,6Gal moieties and the serum levels of the clusterin having this glycopeptide structure decrease significantly in RCC patients when compared with healthy controls. Our results demonstrate that integrating the bottom-up glycoproteomics with top-down glycopeptidomic approach enables confident validation, in both the structural identification and accurate quantitation of the intact glycopeptides pre-determined by the existing methodologies.

## Results

**Fig. 2:**
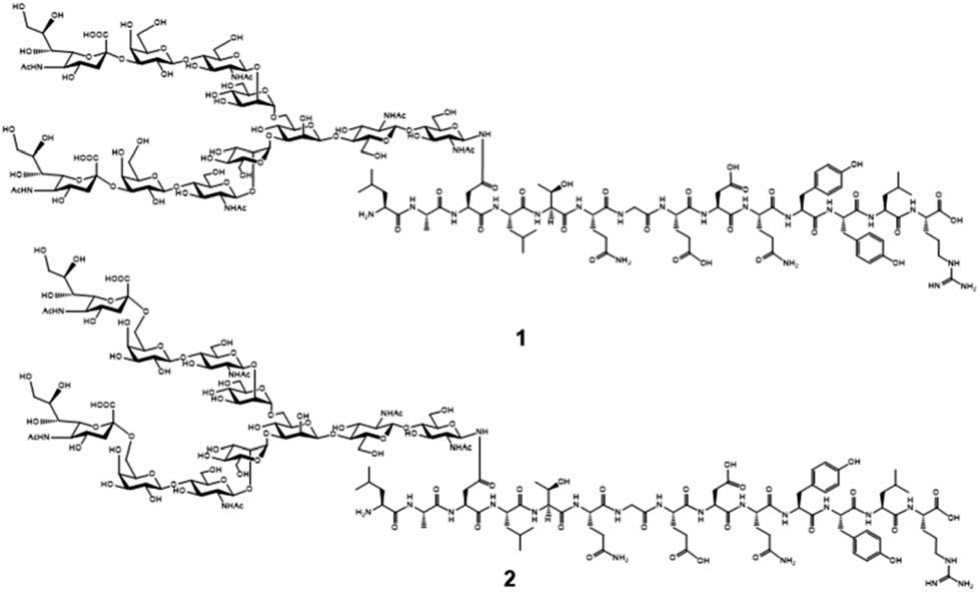
Proposed chemical structures of the targeted clusterin tryptic glycopeptide variants having biantennary disialyl *N*-glycan isomers (A2G2S2) at the Asn374 residue. Glycopeptide 1 is modified with (Neu5Acα2,3Gal)2GlcNAc2Man3GlcNAc2 and its regiospecific isomer 2 bears (Neu5Acα2,6Gal)2 GlcNAc2Man3GlcNAc2.

**Scheme 1:**
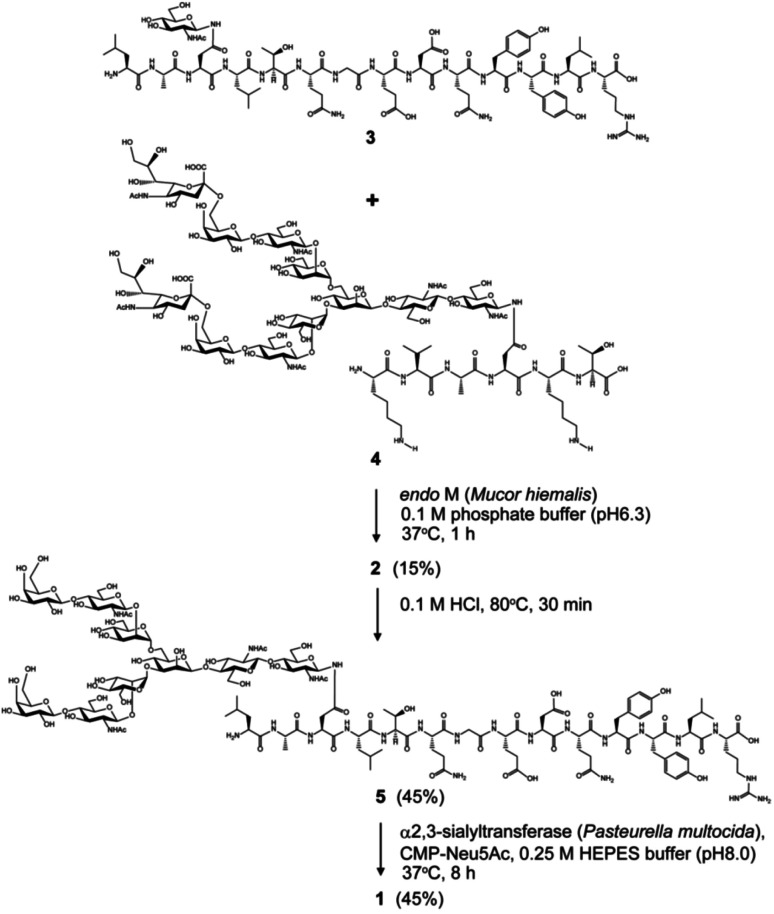
Chemical and enzymatic synthesis of clusterin tryptic glycopeptide variants 1 and 2 from the glycopeptide substrate 3.

**Fig. 3:**
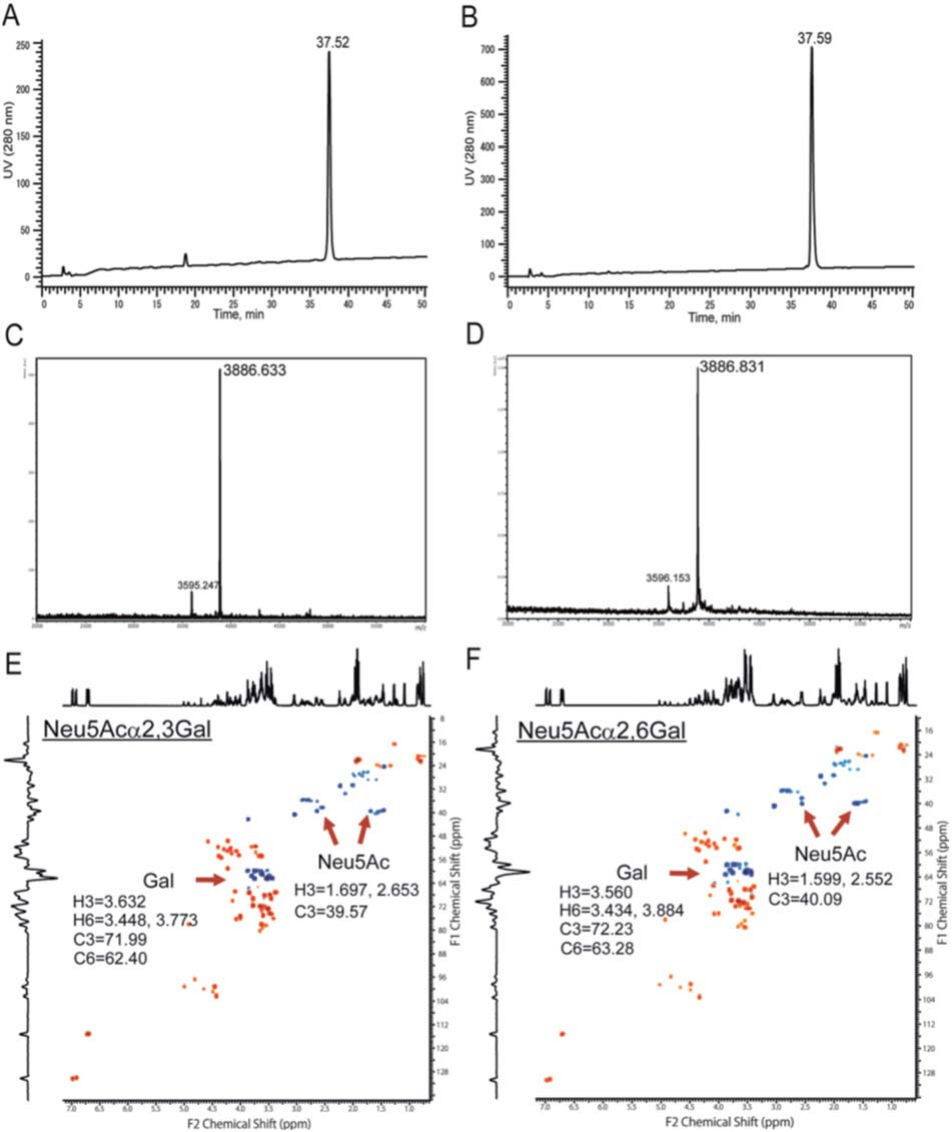
Characterization of the synthetic clusterin glycopeptides 1 and 2. Reverse-phase HPLC profiles of 1 (A) and 2 (B) under the gradient from acetonitrile/H_2_O = 97/3 (0 min) to acetonitrile/H_2_O = 70/30 (50 min), MALDI-TOFMS of 1 (C) and 2 (D) (*m*/*z* 3886.59 calculated for C_158_H_250_N_26_O_86_ [M − H]^−^), and 600 MHz HSQC spectra of 1 (E) and 2 (F) measured in D_2_O at 298 K, respectively. The peaks at *m*/*z* 3595.247 (C) and *m*/*z* 3596.153 (D) are due to the ions having a monosialyl *N*-glycan generated from 1 and 2 by the partial cleavage of one of the terminal sialic acids under the high-energy laser irradiation during MALDI-TOFMS measurements.

Chemical and enzymatic synthesis of cancer-relevant clusterin glycopeptide variants. To test our hypothesis that the SRM assay using synthetic clusterin glycopeptides as calibration standards enables direct structural determination and accurate quantitation of the targeted clusterin glycopeptide fragments carrying an exact glycoform existing in highly complex tryptic digests of sera, we synthesized the clusterin glycopeptide variants having a biantennary disialyl *N*-glycan, (Neu5Acα2,3Gal)_2_GlcNAc_2_Man_3_GlcNAc_2_ (1) and (Neu5Acα2,6Gal)_2_GlcNAc_2_Man_3_GlcNAc_2_ (2), notably isomers generated by regiospecific sialylation (Fig. 2). Scheme 1 represents a synthetic route of the glycopeptides 1 and 2 from a key acceptor substrate 3^372^Leu-Ala-Asn-Leu-Thr-Gln-Gly-Glu-Asp-Gln-Tyr-Tyr-Leu-Arg^385^ bearing a GlcNAc moiety at Asn374. The glycopeptidic acceptor 3 was prepared by solid-phase synthesis using Fmoc-amino acid derivatives and Fmoc-Asn(Ac_3_GlcNAc)OH on a Trityl-Chem matrix resin under a microwave-assisted method according to the conditions reported previously,^28,29,33,35^ and it was subsequently employed for the enzymatic *trans*-glycosylation with a commercially available donor substrate 4, egg yolk-derived sialoglycopeptide (SGP) having a biantennary disialyl *N*-glycan (Neu5Acα2,6Gal)_2_GlcNAc_2_Man_3_GlcNAc_2_. The *trans*-glycosylation reaction between 3 and 4 catalysed by recombinant *endo*-β-*N*-acetyl-d-glucosaminidase (*endo*-M from *Mucor hiemalis*)^36^ proceeded smoothly while substantial amounts of hydrolytic products from donor 4 afforded the target glycopeptide 2 in 15% isolated yield (Fig. S1†). To replace the glycoside linkage between sialic acid and galactose residue of the compound 2 from Neu5Acα2,6Gal into Neu5Acα2,3Gal, the asialo-glycopeptide intermediate 5, derived readily by treating 2 under mildly acidic conditions was subjected to re-sialylation by using a bacterial α2,3-sialyltransferase (*Pasteurella multocida*)^37^ in the presence of cytidine-5′-monophospho-*N*-acetylneuraminic acid sodium salt (CMP-Neu5Ac), giving rise to the targeted clusterin isomeric glycopeptide 1 in 45% isolated yield. The purity and chemical structures of synthetic glycopeptides 1 and 2 were elucidated by reverse-phase HPLC, MALDI-TOFMS, and 1H-/13C-NMR (HSQC) spectra shown in Fig. 3. Characterization data of all new compounds 1, 2, 3, and 5 synthesized herein are also described in the ESI (Fig. S1–S4 and Table S1†).

SRM channel setting by using synthetic clusterin glycopeptides. Optimization of SRM channel setting by using synthetic clusterin glycopeptides 1 and 2 was performed as follows: (a) precursor ion selection at the first quadrupole (Q1), (b) collision-induced dissociation (CID) in the second quadrupole (Q2), and (c) specific fragment ion scanning in the third quadrupole (Q3), commonly referred to as transitions. The quadrupole works as a mass filter and excludes other ions except the target ion, implying that fragmentation can be optimized by monitoring the actual measurement values obtained only from the ions corresponding to the synthetic clusterin glycopeptide 1 or 2. In addition, we may optimize LC functions such as solvents and gradient to separate elution times of the isomeric targets. SRM parameters for the glycopeptides 1 and 2 were optimized by using the 4000Q Trap triple quadrupole mass spectrometer with UltiMate 3000 HPLC (Table S2†).

**Fig. 4:**
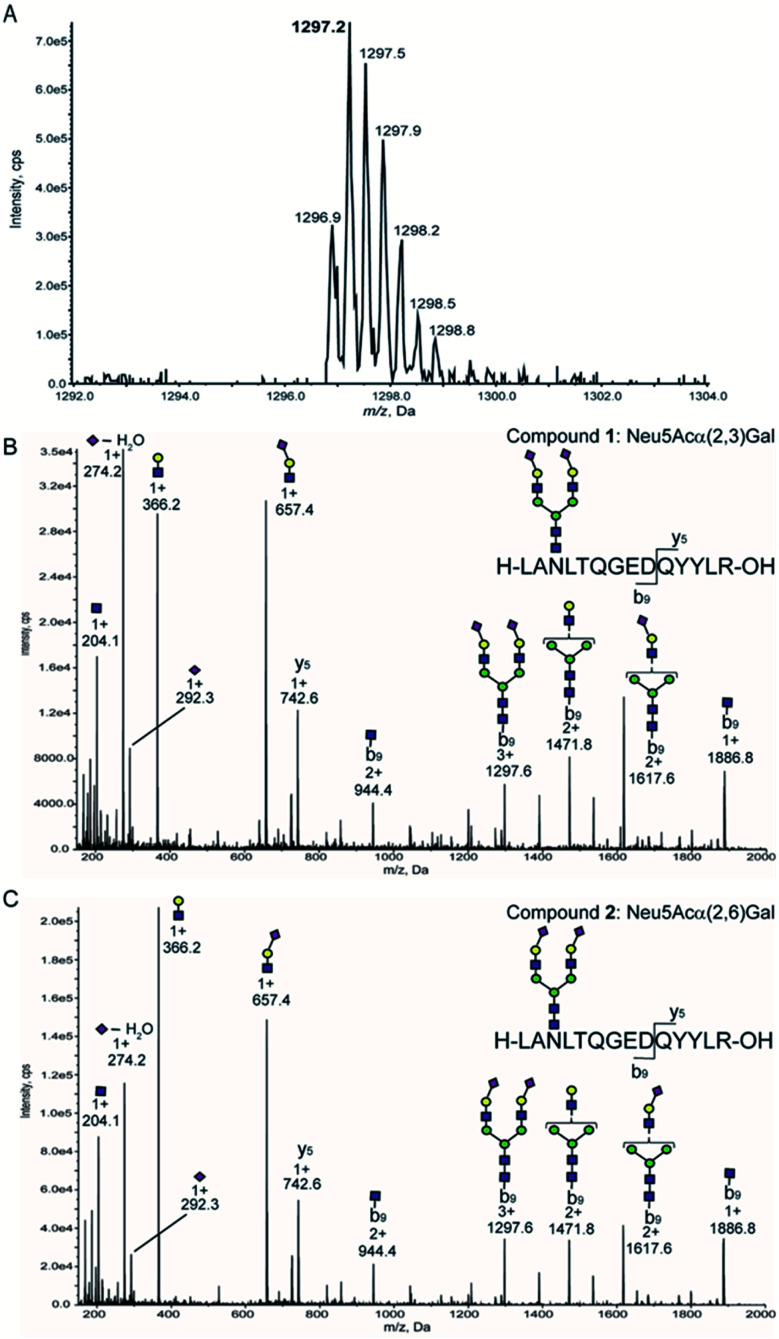
Optimization of SRM channels by structure-defined synthetic clusterin glycopeptides. (A) MS spectrum of glycopeptide 1 measured by the enhanced resolution mode. MS/MS fragmentation patterns generated from isomeric glycopeptides 1 having Neu5Acα2,3Gal terminals (B) and 2 having Neu5Acα2,6Gal terminals (C), respectively.

Given that precursor ions (Q1) from both glycopeptides 1 and 2 were detected as *m*/*z* 1297. 2 centred, with the isotopomer-difference of 0.3 Da as shown in Fig. 4A, they can be confirmed to be ionized as proton adduct trivalent positive 3 + ions, *m*/*z* 1297.21 calculated for [C_158_H_250_N_26_O_86_ + 3H]^3+^. Then, we measured the fragmentation patterns of the precursor ions by CID with the gradual increase of the collision energy from 25 volts (0.8 min) to 130 volts (5 min) at Q2. It is interesting to note that the CID of precursor ions for glycopeptides 1 and 2 generated clearly different profiles of the fragment ions under the same conditions as shown in Fig. 4B and C. The relative intensities corresponding to the dehydrated Neu5Ac oxonium ion (*m*/*z* 274.2, [C_11_H_18_NO_8_]^+^) produced by the cleavage at the glycoside bond between the Neu5Ac and Gal residues appeared to be different between compounds 1 and 2 in comparison to those of other fragment oxonium ions such as sialyl LacNAc (*m*/*z* 657.4, [C_25_H_41_N_2_O_18_]^+^), LacNAc (*m*/*z* 366.2, [C_14_H_24_NO_10_]^+^), and GlcNAc (*m*/*z* 204.0, [C_8_H_14_NO_5_]^+^).

Glycopeptide 1 provided a significantly higher level of this fragment ion when compared with that from glycopeptide 2, suggesting that the glycoside linkage of the Neu5Acα(2 → 3)Gal moiety may be more sensitive and labile to the CID condition than the Neu5Acα(2 → 6)Gal structure of the glycopeptide 2. Indeed, our previous study indicated that the difference in the conformational stabilities between Neu5Acα(2 → 3)Gal and Neu5Acα(2 → 6)Gal structures provides distinctively characteristic features of the tandem mass spectrometry MS^n^ fragmentation patterns.^38^ Importantly, four carbohydrate oxonium-type fragment ions liberated from both glycopeptides 1 and 2 showed significantly higher intensities than those of the ions due to the peptide-type fragments, notably y5 (*m*/*z* 742.6) and its partner ions detected at *m*/*z* 944.4, *m*/*z* 1297.6, *m*/*z* 1471.8, *m*/*z* 1617.6, and *m*/*z* 1886.8. These results clearly demonstrate that highly sensitive sugar oxonium ions are ideal Q3 candidates for the SRM-based quantitation of clusterin tryptic glycopeptides 1 and 2 without the influence of other peptide-containing fragment ions detected in the range from *m*/*z* 742.6 to *m*/*z* 1886.8.

Table 1 summarizes the potential MS/MS channels optimized for the clusterin tryptic glycopeptides 1 and 2 based on the four-carbohydrate oxonium-type fragment ions. It is interesting to note that sialyl LacNAc oxonium ion (*m*/*z* 657.4) can be produced by the lowest CE (35 V) among these four-carbohydrate-type oxonium ions, while the generation of GlcNAc oxonium ion needs the highest CE (73 V).

**Table 1:** Potential SRM channels optimized for the clusterin glycopeptides 1 and 2^a^

Fragment oxonium ion	Q1	Q3	DP	EP	CE	CXP
GlcNAc	1297.2	204.0	121	10	73	14
Dehydrated Neu5Ac	1297.2	274.2	126	10	69	14
LacNAc	1297.2	366.2	121	10	58	14
Sialyl LacNAc	1297.2	657.4	121	10	35	14

a DP: declustering potential (V), EP: entrance potential (V), CE: collision energy (V), CXP: collision cell exit potential (V).

**Fig. 5:**
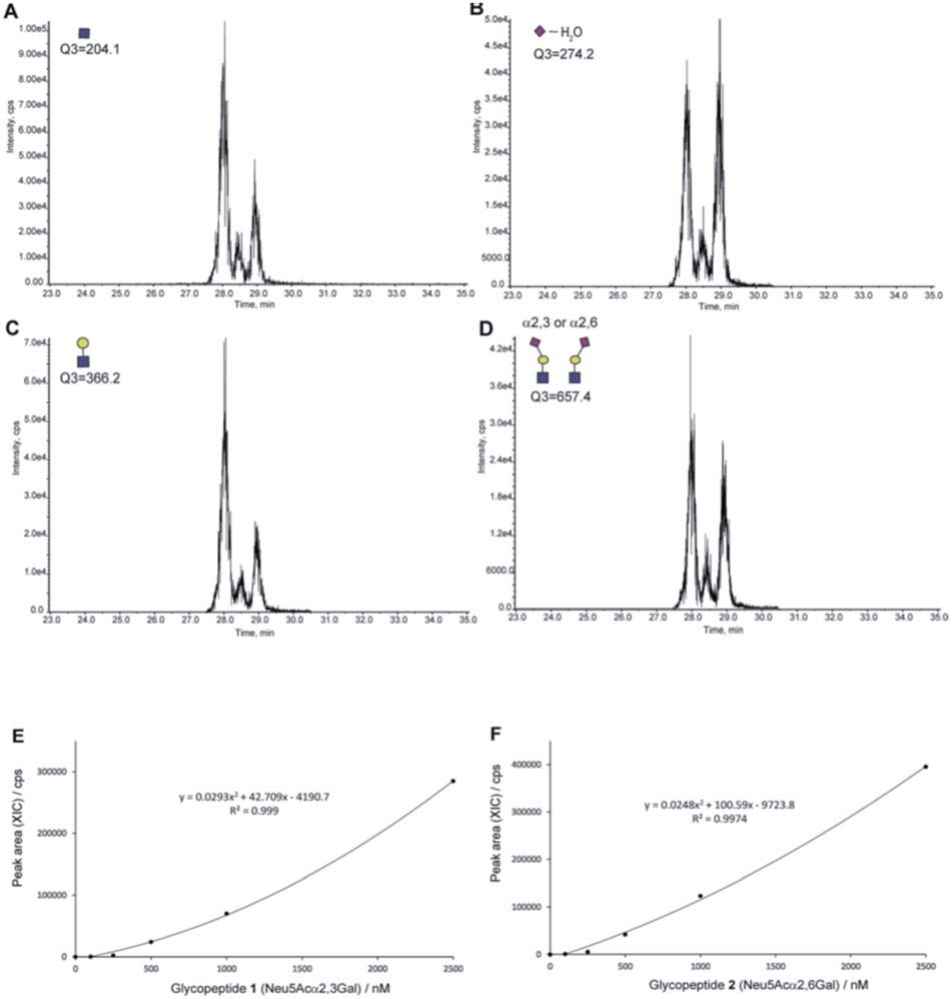
The SRM channels (four transitions) allowing the discriminative quantitation of isomeric clusterin glycopeptides 1 and 2. XICs of the standard solution [an equal volume (each 5 µL) of synthetic clusterin glycopeptides 1 and 2 (each 25 pmole)] measured by SRM channels (Q1/Q3) for (A) the GlcNAc oxonium ion (1297.2/204.0), (B) dehydrated Neu5Ac oxonium ion (1297.2/274.2), (C) LacNAc oxonium ion (1297.2/366.2), and (D) sialyl LacNAc oxonium ion (1297.2/657.4), respectively. Calibration curves of glycopeptides 1 (E) and 2 (F) by means of the SRM channel for the sialyl LacNAc oxonium ion (Q1/Q3 = 1297.2/657.4). XICs at 28.16 ± 0.07 min (2) and 29.09 ± 0.03 min (1) were measured by using the standard solutions (0, 100, 250, 500, 1000, and 2500 nM) containing synthetic clusterin glycopeptides 1 and 2 (Fig. S6, Tables S3 and S4†). LC conditions [flow rate: 200 µL min^−1^, gradient: (a) 0.1% formic acid in water/(b) 0.1% formic acid in acetonitrile, (a)/(b) = 90/10 at 0 min → 75/25 at 45 min].

Next, we tested the feasibility of the four SRM channels Q1/Q3 (1297.2/204, 1297.2/274.2, 1297.2/366.2, and 1297.2/657.4) by measuring the extracted ion chromatograms (XICs) of a mixture composed of an equal volume (5 µL each) of the synthetic clusterin glycopeptides 1 and 2 (each 25 pmol) under the optimized LC conditions to separate these isomers [flow rate: 200 µL min^−1^, gradient: (A) 0.1% formic acid in water/(B) 0.1% formic acid in acetonitrile, (A)/(B) = 90/10 at 0 min → 75/25 at 45 min].^39^ As shown in Fig. 5A–D, all XICs measured by four SRM channels gave two distinct peaks at 28.11 and 29.09 min corresponding to the two-isomeric glycopeptides 1 and 2, indicating that these Q1/Q3 sets could be used for the quantitation of these two isomers, concurrently. Judging from the characteristic features of the XICs (Fig. 5), particularly the distinct XIC profiles obtained when the SRM assay was conducted by using Q1/Q3 (1297.2/274.2) that generates efficiently dehydrated Neu5Ac oxonium ion (Table 1), we concluded that the peak detected at 29.09 min corresponds to glycopeptide 1 having two Neu5Acα2,3Gal terminals. Notably, it was indicated that the intensity of the dehydrated Neu5Ac oxonium ion derived from the Neu5Acα2,3Gal moiety is remarkably higher than that from the isomeric Neu5Acα2,6Gal structure under the same MS/MS conditions when compared with other fragment ions (Fig. 4B and C). In contrast, production of the dehydrated Neu5Ac oxonium ion observed at 28.11 min (Fig. 5B) was relatively lower as compared to other fragment ions (Fig. 5A, C, and D) when compared with the results observed at 29.09 min, suggesting that another major peak at 28.11 min is due to glycopeptide 2 having two Neu5Acα2,6Gal terminals. Of note, it seems likely that a small peak detected between the two major peaks might be due to an extremely small amount of the glycopeptide with a bi-antennary *N*-glycan containing both isomer Neu5Acα2,6Gal and Neu5Acα2,3Gal (heterogeneous) terminals generated during the enzymatic modification using a bacterial (*Pasteurella multocida*) sialyltransferase as reported previously.^37^

Given the results that sialyl LacNAc oxonium ion (*m*/*z* 657.4) produced by fragmentation from synthetic glycopeptides 1 and 2 needs the lowest CE (35 V) among four CEs (Table 1), we employed SRM channel Q1/Q3 (1297.2/657.4) for further experiments toward the quantitation and structural determination of the serum clusterin tryptic glycopeptides. Fig. 5E and F represent the calibration curves designated for the SRM-based quantitative analysis of the intact clusterin tryptic glycopeptides corresponding to compounds 1 and 2 (see also, Fig. S5, S6, Tables S3 and S4†), indicating that the calibration curves can be used in the concentration range between approximately 250 and 1000 nM.

Structural identification and absolute quantitation of cancer-associated intact clusterin glycopeptides. The optimized SRM channel using sialyl LacNAc oxonium ion as a transition (*m*/*z* 657.4) that enables the discriminative quantitation of the isomeric clusterin glycopeptides 1 and 2 was applied for the quantitative analysis of human serum clusterin containing the focused glycopeptide structures. However, the comprehensive, and quantitative analysis of human serum/plasma shows a substantial challenge due to the sample complexity and wide dynamic range of glycoprotein concentration. Human serum represents the most complex biological sample of the glycoproteome, composed of more than 10^6^ different glycoproteins, whereas 30 of the most abundant (glyco)proteins constitute approximately 99% of the total protein mass.^40^ The serum concentration of the human clusterin has been reported to be a considerably wide range from 50 to 340 µg mL^−1^ (0.59–4.0 µM),^15,16^ suggesting that this disparity found in the serum clusterin concentration may be due to its potent adhesive properties resulting in the technical difficulties with assay systems. To avoid the influence of many abundant serum proteins in the non-specific binding with clusterin and ion suppression effect by the large amount of tryptic peptides derived from themselves, we established an experimental workflow that enables the depletion of the 14 most abundant serum proteins, namely human serum albumin, IgG, IgM, IgA, haptoglobin, transferrin, alpha 1-antitrypsin, alpha 2-macroglobulin, complement C3, alpha 1-acid glycoprotein 1, apolipoprotein AI, transthyretin, apolipoprotein AII, fibrinogen beta chain, fibrinogen alpha chain, and fibrinogen gamma chain, by multiple affinity depletion using MARS 14 immunodepletion cartridge.^41,42^ After removal of the salts, lipids, and low molecular weight substances, depleted sera were subjected to reductive alkylation, tryptic digestion, and SRM assay.

**Fig. 6:**
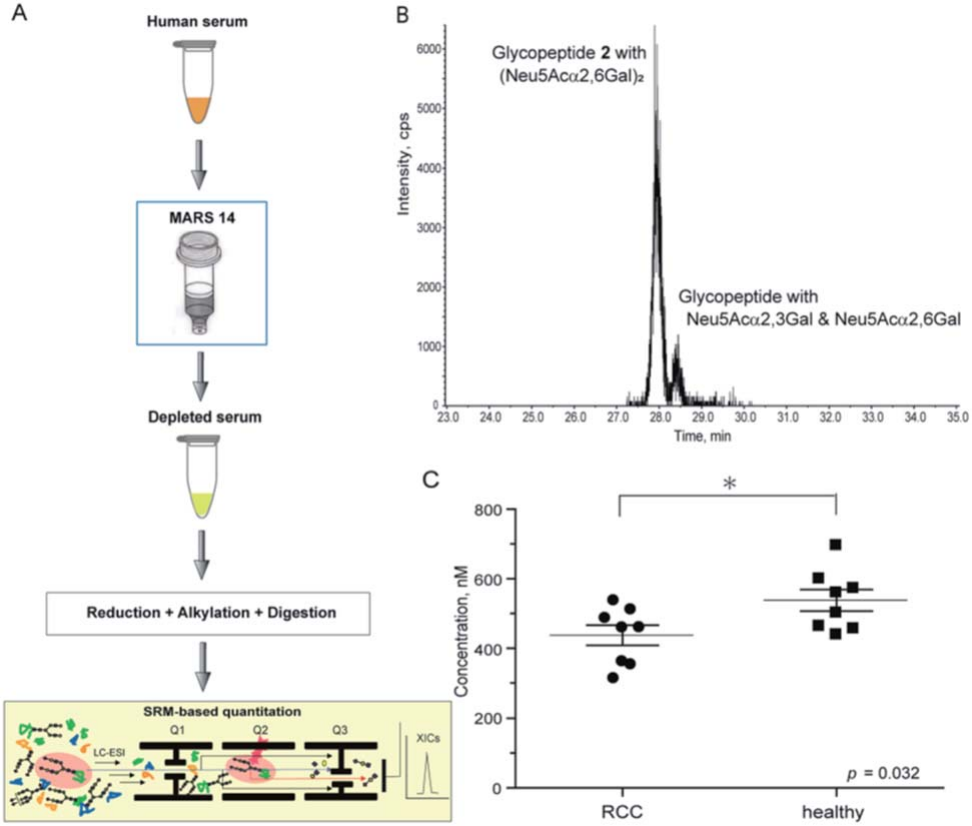
SRM-based absolute quantitation of human serum clusterin having the targeted glycopeptide 2. (A) An experimental workflow for the human serum sample preparation (see ESI, Experimental†). (B) An example of the XIC, a major peak observed at 28.15 min (healthy control, entry 11) provides evidence that human serum clusterin contains dominantly isomeric glycopeptide moiety 2 having a homo sialyl *N*-glycan with two Neu5Acα2,6Gal terminals while a small peak detected at ∼28.5 min indicates existence of the modification by a hetero sialyl *N*-glycan with Neu5Acα2,3Gal and Neu5Acα2,6Gal terminals at the Asn374 residue. (C) Absolute concentration of the clusterin-derived tryptic glycopeptide 2 in the depleted sera from RCC patients (*n* = 8) and healthy controls (*n* = 8), respectively (see the ESI Table S5†).

Using this experimental workflow (Fig. 6A), we succeeded in the SRM-based absolute quantitation of the intact clusterin glycopeptides derived from the sera of 16 individuals, including 8 individuals with RCC and 8 control individuals (Table S5†). A typical XIC (Fig. 6B) and all other results (Fig. S7†) clearly showed the occurrence of the highest peak at 28.17 ± 0.26 min corresponding to the sialyl LacNAc oxonium ion (*m*/*z* 657.4) derived from clusterin tryptic glycopeptide 2 (Fig. 5A–D), demonstrating that circulating human clusterin is glycosylated at Asn374 dominantly by a biantennary sialyl *N*-glycan with homo Neu5Acα2,6Gal terminals. Notably, a small peak detected at ∼28.7 min represents the existence of low levels of the hetero sialyl *N*-glycan having both Neu5Acα2,3Gal and Neu5Acα2,6Gal terminals in 16 individuals tested while the peak at ∼29.1 min corresponding to the sialyl LacNAc oxonium ion from isomeric glycopeptide 1 (Fig. 5A–D) was detected exceptionally only in case of one RCC patient (Fig. S2(D)†), indicating that modification by homo sialyl *N*-glycan with Neu5Acα2,3Gal terminals is not common at Asn374 residue of the human clusterin.

As shown in Fig. 6C (Fig. S7 and Table S5†), the SRM assay using the calibration curves for the designated synthetic glycopeptides 1 and 2 allowed, for the first time, top-down glycopeptidomics for both the structural confirmation and absolute quantitation of clusterin sialylated glycopeptide isomers in the range from 313.3 to 697.5 nM (12.2–27.2 ng µL^−1^) in highly complex tryptic digests of the depleted serum samples from RCC patients and healthy controls. Concentrations (Mean ± SEM, standard error of mean) of the clusterin tryptic glycopeptide 2 from the RCC patient serum (437.2 ± 29.0 nM) were found to be significantly lower than those from the serum of healthy subjects (537.9 ± 30.9 nM) (*p* = 0.032). These results indicate that the circulating clusterin modified at Asn374 by a biantennary sialyl *N*-glycan with homo Neu5Acα2,6Gal terminals is approximately 20% downregulated in RCC tumour patients as compared to healthy controls.

## Discussion

The results unveiled, for the first time, that circulating human clusterin is modified at Asn374 dominantly by a biantennary sialyl *N*-glycan with homo Neu5Acα2,6Gal terminals. Serum concentrations of the clusterin intact glycopeptide 2 with homo Neu5Acα2,6Gal terminals derived from the RCC patient serum (437.2 ± 29.0 nM) were found to be at significantly lower levels than those derived from the healthy subjects (537.9 ± 30.9 nM), indicating that circulating clusterin containing the structure corresponding to glycopeptide 2 is decreased by approximately 20% in the RCC patients' group as compared to healthy controls (Fig. 6). Our current observations are in a similar propensity with the results reported previously by the Hancock and Iliopoulos group that plasma levels of clusterin having a biantennary disialyl *N*-glycan (A2G2S2 or FA2G2S2) at Asn374 residue are reduced relatively in patients before RCC(+) as compared with after curative nephrectomy RCC(−).^24^ These results may imply that (a) the biantennary disialyl *N*-glycans (A2G2S2 and/or FA2G2S2) with homo Neu5Acα2,6Gal terminals at Asn374 residue have an important role in the antitumor activity of the clusterin in the healthy group and patients after curative nephrectomy RCC(−), or (b) the triantennary trisialyl *N*-glycans increased inversely at this site^24^ are beneficial for cancer cells to evade the anti-cancer immune response. It is interesting to speculate that the site-specific attachment of the triantennary trisialyl *N*-glycans at Asn374 residue can provide clusterin with enhanced affinity for Siglecs on myeloid cells (monocytes) inducing tumour-associated macrophages.^43–46^ To test this new hypothesis, we plan to synthesize clusterin glycopeptide variants having a triantennary trisialyl *N*-glycan with regio-isomeric structures such as homo Neu5Acα2,3Gal, homo Neu5Acα2,6Gal, and possible hetero Neu5Acα2,3Gal/Neu5Acα2,6Gal terminals^47,48^ at the Asn374 residue and determine the RCC patients' serum concentrations of the clusterin containing these glycoforms at Asn374 by the SRM-based top-down glycopeptidomic approach.

**Fig. 7:**
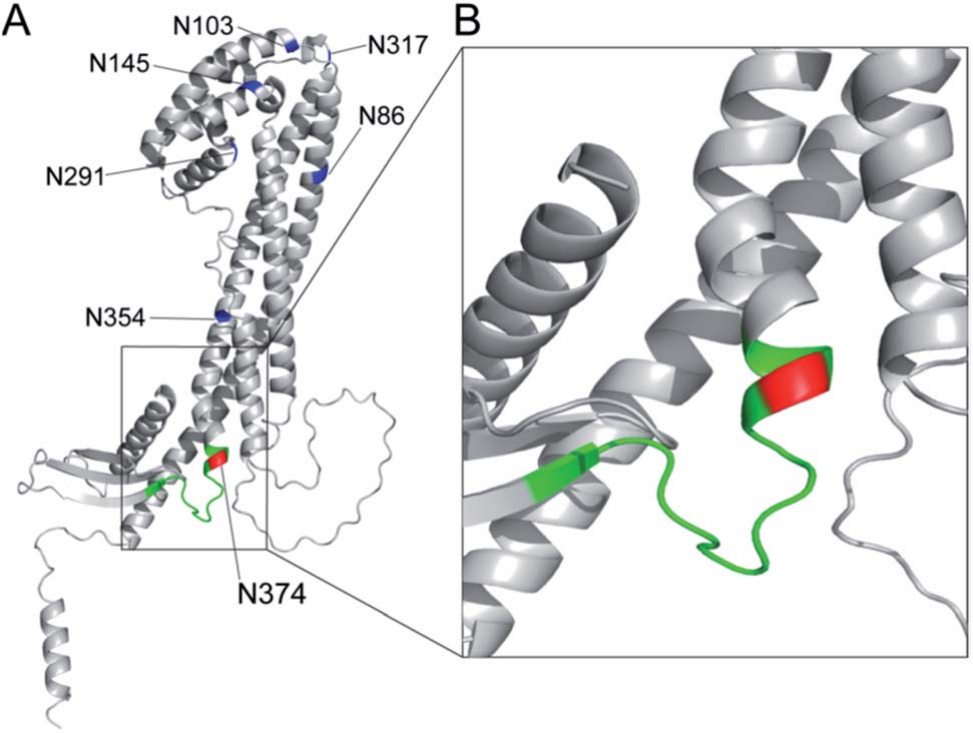
Three-dimensional structure of human clusterin predicted by the neural network AlphaFold^49^ (structure prediction last updated on December 9, 2021 with AlphaFold Monomer v2.0) cited from AlphaFold Protein Structure Database (https://alphafold.ebi.ac.uk/entry/P10909). (A) Locations of seven potential *N*-glycosylation sites in human clusterin are represented by blue for Asn86, Asn103, Asn145, Asn291, Asn317, and Asn354, and by red for the Asn374 residue. The region shown by green indicates the tryptic peptide moiety, ^372^Leu-Ala-Asn-Leu-Thr-Gln-Gly-Glu-Asp-Gln-Tyr-Tyr-Leu-Arg^385^. (B) An enlarged view focusing on the structure including the Asn374 residue and its neighbouring area.

The three-dimensional structure of human clusterin predicted by the neural network AlphaFold^49^ uncovered the distinctive location and conformation of the peptide sequence from Leu372 to Arg385, including the glycosylated Asn374 residue (Fig. 7A). Surprisingly, the clusterin tryptic glycopeptide containing the Asn374 residue seemed to be located in the unstructured long loop-like region between the α-helix and anti-parallel β-sheet structures (Fig. 7B). In contrast, regions containing Asn86 and Asn354 appeared to be involved entirely in the well-folded α-helix structure, and 4 other potential glycosylation sites, Asn103, Asn145, Asn291, and Asn317, were also distributed mostly in the tight junction area between the two α-helix structures (Fig. 7A). It is likely that site-specific modification at the Asn374 residue may provide glycans attached to this site with much higher flexibility than other potential glycosylation sites in the clusterin, while the conformational impact of the glycosylation on each of the peptide regions remains. Further systemic structural and biochemical studies are required for insight into the significance of the site-specific glycosylation status in the anti-tumour and immunosuppressive effect of clusterin on various cancer cells.

Accumulating results provide evidence that site-specific protein *N*- and *O*-glycosylation often induces dynamic conformational alterations in the proximal peptide area including the amino acid residue to be glycosylated in a peptide sequence-dependent manner.^50–59^ Our studies have revealed that conformational impact by *O*-glycosylation at the immunodominant peptide motifs of cancer-associated mucin glycoproteins is strongly affected by the cancer-specific glycoforms, resulting in the generation of unique glycopeptidic neoepitopes, namely “dynamic epitopes”, as potential targets for the development of diagnostic and therapeutic mAbs.^10–12,56,58^ Recently, we established a generalizable strategy that allows for the creation of mAbs interacting specifically with such dynamic epitopes by using a set of homogeneous synthetic glycopeptide derivatives as key materials for the immunization, antibody screening, epitope mapping, and biochemical/structural analysis.^12,60,61^

**Fig. 8:**
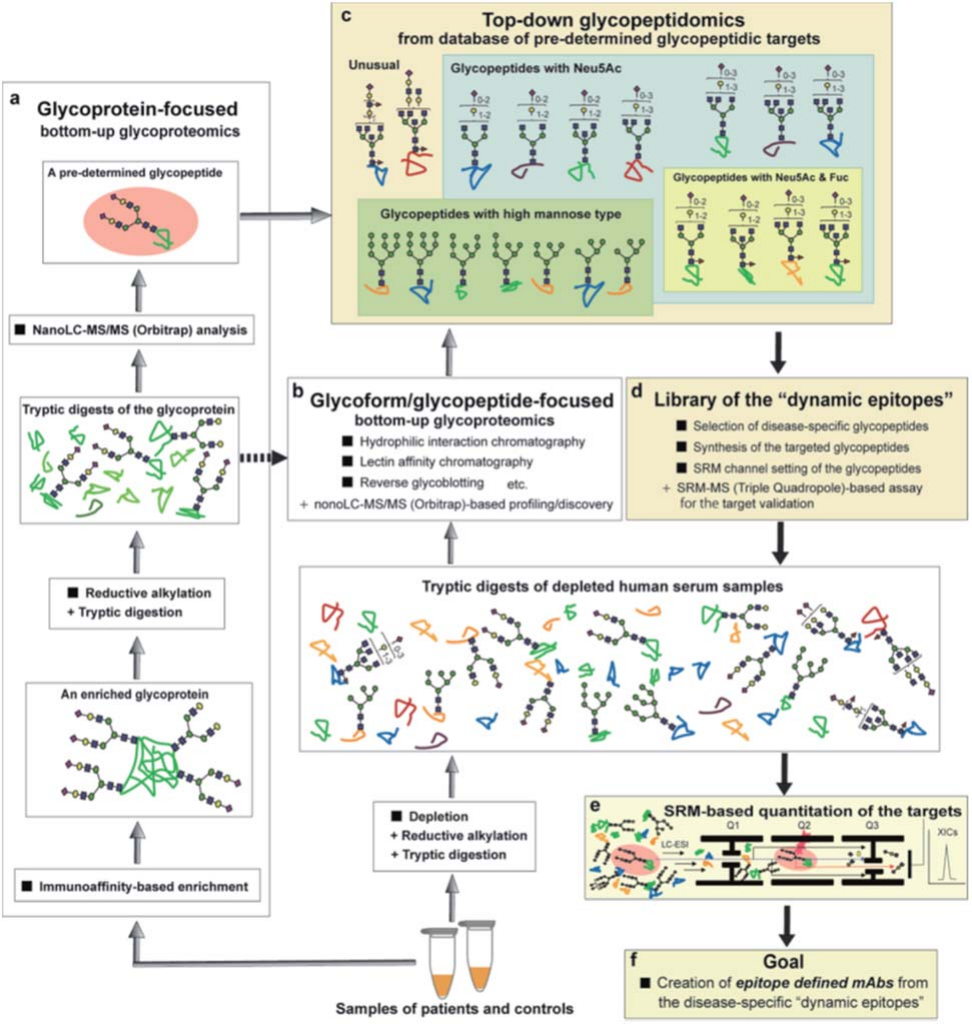
Overview of a new strategy for the development of disease-specific glycopeptidic biomarkers from human serum samples by integrating bottom-up glycoproteomics with the top-down glycopeptidomic approach. Pre-determined glycopeptidic targets can be provided by both glycoprotein-focused (a) and glycoforms/glycopeptide-focused (b) glycoproteomic protocols (grey arrows), leading to the construction of a database for the candidates of disease-specific glycopeptidic biomarkers and a concept of “top-down glycopeptidomics” indicated by black arrays (c). Selected targetable molecules of interest are synthesized systematically, subjected to the SRM channel setting and the target validation, and filed in the library of “dynamic epitopes” (d). The target validation is performed by SRM-based accurate quantitation using the tryptic digests derived from sera of patients and normal controls (e). The dynamic epitopes showing clinically potent characteristics as diagnostic and therapeutic targets may be employed for the development of new classes of epitope-defined mAbs (f).

As shown in an overview of the novel strategy to integrate the bottom-up glycoproteomics with top-down glycopeptidomics (Fig. 8), a variety of pre-determined glycopeptides as potential candidates for the dynamic epitopes will be provided both by the glycoprotein-focused and glycoform/glycopeptide-focused bottom-up glycoproteomic protocols. The clusterin tryptic glycopeptide including the Asn374 residue was initially identified by using immunoaffinity-based enrichment analysis of the disease-associated clusterin,^24^ indicating the merits of a functionally “known” glycoprotein-focused bottom-up glycoproteomics. On the other hand, the glycoform/glycopeptide-focused glycoproteomic approach, notably glycopeptide enrichment analysis, is a potential approach for the discovery of “unknown” glycopeptidic biomarkers that can discriminate between patients and healthy individuals. However, heterogeneities produced both by the glycan occupancy at multiple sites of a protein (macro-heterogeneity) and the variable number and levels of glycoforms at one or more glycosylation sites in the protein (micro-heterogeneity) have largely hampered systemic glycoproteomic studies.

The advantage of the top-down glycopeptidomics for validating pre-determined glycopeptidic biomarkers provided by the bottom-up glycoproteomics is evident because the SRM-based approach using structure-defined synthetic glycopeptides concurrently enables accurate structure identification and reliable quantitation of the targeted label-free tryptic glycopeptides derived from complex biological samples.

## Conclusions

The top-down glycopeptidomics based on the SRM approach using synthetic clusterin glycopeptides provided evidence that alteration of the site-specific glycosylation at Asn374 of the human clusterin found by Hancock and Iliopoulos team is due to the decrease of the biantennary sialyl *N*-glycan with homo Neu5Acα2,6Gal terminals, whereas there is no isomeric conversion from the Neu5Acα2,6Gal into the Neu5Acα2,3Gal structure during the progression of RCC. This study demonstrated that a new strategy integrating the bottom-up glycoproteomics with top-down glycopeptidomics using structure-defined synthetic glycopeptides enables confident structural identification and absolute quantitation of the label-free glycopeptidic targets pre-determined by the existing methods for intact glycopeptide profiling. Considering that the number of candidates for the pre-determined disease-specific glycopeptides may be increased rapidly by the improvement of technologies in the bottom-up glycoproteomic approach, a compound library of the synthetic glycopeptides (a set of peptides with glycan microheterogeneity) with each SRM channel would accelerate top-down glycopeptidomics for the discovery of new “dynamic epitopes” as ideal disease-specific glycopeptidic targets as outlined in Fig. 8.

## Experimental

### Synthesis of clusterin glycopeptides

Microwave-assisted solid-phase syntheses and systemic enzymatic manipulation of clusterin glycopeptides 1 and 2 were performed according to the methods reported previously.^28,29,33,35^ Details on the synthetic conditions and characterisation data of all new compounds are described in the ESI.†

### NMR analysis

^1^H NMR and HSQC spectra for the structural characterization of the synthetic glycopeptides 1, 2, 3, and 5 were measured with a 600 MHz instrument and recorded at 298 K (Fig. S1–S4†). Based on the obtained data, the structures of all new compounds were confirmed by attributing each proton and carbon as summarized in Table S1.† The chemical shifts of each amino acid residue in the peptide backbone can be seen to change by the regio-selective sialylation of the biantennary *N*-glycans. The chemical shifts of Neu5Ac and Gal residues also differed between Neu5Acα2,3Gal and Neu5Acα2,6Gal structures (Fig. 3).

### Serum sample preparation

The procedures for high-abundant protein depletion using MARS,^41,42^ reductive alkylation, and tryptic digestion of human serum samples are described in the ESI.†

### LC-MS/MS modes and conditions

SRM analysis by LC-MS/MS was conducted with Dionex HPLC and AB Sciex 4000Q Trap® TurboIonSpray systems. Separation was performed with an LPG-3x00 pump, WPS-3000 auto sampler, FLM-3100 column component, and WVD-3400 detector under the control of the software Chromeleon 6.80 on Inertsil ODS-3 (2.1 × 150 mm, GL Sciences Inc). All data were analyzed by a series of software, Analyst 1.5 and MultiQuant 1.1.0.26. In MS/MS and LC-MS/MS analyses, measurements were performed by appropriate modes under the recommended default conditions as follows: (a) determination of the Q1 channel, enhanced mass mode and enhanced resolution mode; (b) determination of Q2 parameters, compound optimization mode; (c) determination of Q3 channels, enhanced product ion mode; (d) SRM/MRM assay, scheduled MRM mode; (e) LC conditions, multi-step gradient [(A) 0.1% FA aq. (B) 0.1% FA in CAN, 0 min: (A)/(B) = 90/10 → 45 min: 75/25 → 46 min: 10/90 → 50 min: 10/90 → 50.1 min: 90/10 → 60 min: 90/10]; column temperature, 25 °C; and injection volume, 10.0 µL. The SRM experiments were performed at a scan time of 0.10 s, dwell time of 20 ms, and peak width of 0.20 FWHM for glycopeptide 1 and 0.21 FWHM for glycopeptide 2.

### Statistical analysis

Statistical analysis was carried out in GraphPad Prism 8.0.2. Quantitative data are presented as mean ± s.d., if not stated otherwise. Differences were compared using the Student's *t*-test. *P* values were 0.05 or less, the differences were considered statistically significant.

## Ethical statement

This study was performed in accordance with the ethical standards of the Declaration of Helsinki and approved by the Institutional Ethics Committee of Hirosaki University Graduate School of Medicine, Hokkaido University Graduate School of Advanced Life Science, and Showa University Department of Laboratory Medicine. Written informed consent was obtained from all serum donors.

## Conflicts of interest

There are no conflicts to declare.

## Supplementary Material

RA-012-D2RA02903K-s001
