# Supplementary material for: Selective reaction monitoring approach using structure-defined synthetic glycopeptides for validating glycopeptide biomarkers pre-determined by bottom-up glycoproteomics
Source: RSC Adv. 2022 Aug 3;12(33):21385–93. doi: 10.1039/d2ra02903k (PMC9347767; doi:10.1039/d2ra02903k)
Supplement: RA-012-D2RA02903K-s001 [file RA-012-D2RA02903K-s001.pdf]

Electronic Supplementary Material (ESI) for RSC Advances.
This journal is © The Royal Society of Chemistry 2022

[Supplementary Information]

Selective reaction monitoring approach using structure-defined synthetic glycopeptides for validating glycopeptide biomarkers pre-determined by bottom-up glycoproteomics

Kouta Shiratori,[1] Yasuhiro Yokoi,[2] Hajime Wakui,[1] Nozomi Hirane,[1] Michiru Otaki,[1] Hiroshi Hinou,[1] Tohru Yoneyama,[3] Shingo Hatakeyama,[3] Satoshi Kimura,[4] Chikara Ohyama,[3] and Shin-Ichiro Nishimura*[1,2]

[1]Graduate School of Life Science and Advanced Life Science, Hokkaido University, N21, W11, Kita-ku, Sapporo 001-0021, Japan

[2]ENU Pharma, Co., Ltd., N7, W6, Kita-ku, Sapporo 060-0807, Japan

[3]Department of Urology, Graduate School of Medicine, Hirosaki University, Hirosaki 036-8562, Japan

[4]Department of Laboratory Medicine and Central Clinical Laboratory, Showa University, Northern Yokohama Hospital, Yokohama 224-8503, Japan

Supplementary Information Page(s)

Experimental		S3-12
Figure S1	NMR spectra of compound **1**	S13
Figure S2	NMR spectra of compound **2**	S14
Figure S3	NMR spectra of compound **3**	S15
Figure S4	NMR spectra of compound **5**	S16
Figure S5	HPLC profiles to determine the concentrations	S17
Figure S6	XICs for the preparation of calibration curves	S18-19
Figure S7	XICs in the SRM assay of human serum samples	S20-21
Table S1	Chemical shifts of glycopeptides **1**, **2**, **3**, and **5**	S22-26
Table S2	Mass parameters used in this study	S27
Table S3	Amino acid analysis of glycopeptide **3**	S28
Table S4	Concentrations of glycopeptides **1** and **2**	S28
Table S5	Serum samples and the results of SRM assay	S29

EXPERIMENTAL

Materials and general methods. This study was performed in accordance with the ethical standards of the Declaration of Helsinki and approved by the Institutional Ethics Committee of Hirosaki University Graduate School of Medicine, Hokkaido University Graduate School of Advanced Life Science, and Showa University Department of Laboratory Medicine. Written informed consent was obtained from all serum donors. Serum samples from newly diagnosed RCC patients (n=8) were collected before treatment at Hirosaki University[S1] and healthy control samples (n=8) were collected at Showa University Hospital, respectively. After serum collection, all samples were immediately aliquoted, frozen, and stored at -80°C until pre-processing by our experimental workflow.

All commercially available solvents and reagents were used without further purification. Trityl-OH-ChemMatrix® resin was purchased from Biotage Japan Ltd. *N*-α-Fluorenylmethoxycarbonyl-amino acid (Fmoc-amino acid) derivatives except for glycosylated amino acids were purchased from Merck Millipore. *N*-α-Fluorenylmethoxycarbonyl-*N*-β-(2-*N*-acetylamido-2-deoxy-β-D-glucopyranosyl)-L-asparagine [Fmoc-Asn(Ac_3β-GlcNAc)-OH] was synthesized by the procedure reported previously[S2] and purchased partly from Medicinal Chemistry Pharmaceuticals, Co. Ltd.

The α2,3-sialyltransferase from *Pasteurella multocida* recombinant, expressed in *E. coli* BL21 were purchased from Sigma-Aldrich. Sialylglycopeptide (SGP) derived from egg yolk and *endo*-β-*N*-acetylglucosaminidase from *Mucor hiemalis* recombinant, expressed in *Candida boidinii* (*endo*-M) were purchased from Tokyo Chemical Industry Co., Ltd. Cytidine-5′-monophospho-*N*-acetylneuraminic acid sodium salt (CMP-Neu5Ac) was purchased from Yamasa Co., Ltd. Sequencing Grade Modified Trypsin was purchased from Promega Corporation.

Mixing operation in peptide synthesis was performed by a vortex mixer. The microwave was irradiated during coupling reactions and Fmoc removal using temperature control at 50°C. MALDI-TOFMS spectra was obtained with Bruker ultraflex III mass spectrometer in reflector positive or linear negative mode using matrix as 2,5-dihydroxybenzoic acid (DHB). Typically, the sample were dissolved in 1 μL of 50% aqueous acetonitrile (ACN) and mixed with the same volume of 10 mg/mL DHB in 50% aqueous ACN containing 0.1% 2,2,2-trifluoroacetic acid (TFA). RP-HPLC analysis was conducted with Hitachi system. Separation was performed with L-6250 intelligent pump, L-7400 UV detector and C18 column: Intersil ODS-3 250×20 mm I.D. (GL Sciences Inc.). Analysis (purity check) was performed with L-7100 pump, L-7405 UV detector, and C18 column: Intersil ODS-3 250×4.6 mm I.D. (GL Sciences Inc.). Solvents used for RP-HPLC

in this study were (a) 0.1% TFA aq. and (b) 0.1% TFA in ACN. All 1H- and 13C-NMR spectra for the structural identification of synthetic glycopeptides were collected with 600 MHz Bruker AVANCE (Bruker Biospin Co.). NMR spectra was recorded at 298 K with ^{1}H: 600 MHz and ^{13}C: 125 MHz. Chemical shift is given in ppm and based on internal tetramethylsilane (TMS): δH 0.00 in $CDCl_3$. NMR spectra is assigned with ACD/NMR processor (Advanced Chemistry Development, Inc.).

Microwave-assisted solid-phase synthesis. Glycopeptide **3**, a key intermediate as an acceptor substrate for *endo*-M, was synthesized according to the procedures reported previously.[S3-S6] The followings are the general protocols and conditions for the solid-phase glycopeptide synthesis assisted by microwave irradiation.[S6] The coupling reactions were performed by using Trityl-ChemMatrix resin (0.41 mmol/g, 76.5 mg, 31.4 μmol), Fmoc-amino acid (125.5 μmol, 4.0 eq.), and Fmoc-Asn(Ac_3β-GlcNAc)-OH (62.7 μmol, 2.0 eq.). Firstly, Trityl-ChemMatrix resin was chlorinated in the following procedure. The resin (76.5 mg) was placed on the bottom of a polypropylene tube equipped with a filter (LibraTube®, Hipep Laboratories) and swollen with dichloromethane (DCM) at room temperature for 2 h. The resin was added 2% $SOCl_2$ in DCM and shaken with vortex mixer for overnight. The resin was washed with DCM six times. Then, the first amino acid derivative, Fmoc-Arg(Pbf)-OH (94.1 μmol, 3.0 eq.) was dissolved in DCM (600 μL)

containing *N*,*N*-dimethylformamide (DMF) (70 μL) and *N*,*N*-diisopropylethylamine (DIEA) (6 eq., 33 μL). This solution was added to the resin and the tube was shaken at room temperature for overnight. After the solution was removed, the resin was added 25% DIEA/methanol and shaken at room temperature for 2 h. Then, the resin was washed with DCM, DMF, methanol, diethyl ether three times each and dried *in vacuo* for overnight. The resin was allowed to swell in DCM for 2 h. After filtration and washed with DMF three times, 20% piperidine in DMF was added to remove Fmoc group and the tube was shaken under microwave irradiation for 3 min. The microwave was irradiated during Fmoc removal and amino acid coupling reactions at 50°C. The resin was washed with DMF three times.

In advance, other Fmoc amino acid derivatives (125.5 μmol, 4.0 eq.) were dissolved in 0.4 M 1-[Bis(dimethylamino)methyliumyl]-1H-benzotriazole-3-oxide hexafluorophosphate (HBTU), 1-hydroxybenzotriazole monohydrate (HOBt)/DMF (4 eq.) and DIEA (188 μmol, 6 eq.) in DMF. Then, the solution of the corresponding Fmoc amino acid derivative was added to the Trityl-ChemMatrix resin displaying the first amino acid residue and mixed under microwave irradiation at 50°C for 9 min. Especially, in case of the reaction with sugar amino acid derivative, Fmoc-Asn(Ac_3β-GlcNAc)-OH (62.7 μmol, 2.0 eq.) was dissolved in 0.4 M HBTU, HOBt/DMF (2 eq.) and DIEA (125

μmol, 4 eq.) in DMF and allowed to react with resin at 50°C for 30 min under microwave irradiation. After washing with DMF three times, unreacted amino groups were capped with acetyl group by treating with a solution (Ac_2O/DIEA/DMF = 85/10/5). The resin was washed with DMF six times. The streamlined procedures such as Fmoc-removal, amino acid coupling, and acetyl capping as mentioned above were performed repeatedly. After completing the total synthesis, cleavage cocktail (TFA: H_2O: triisopropylsilane = 95:2.5:2.5) was added to the resin and the reaction mixture was shaken at room temperature for 2 h. The solution was filtered and TFA was removed by evaporation, and then 10 mL of *t*-butyl methyl ether was added. The filtrate in *t*-butyl methyl ether was centrifuged and supernatant was removed. The residual solid was dissolved in 50% acetonitrile/water and lyophilized. Next, the residue was dissolved in methanol and pH of the solution was adjusted to 12.5 with 1N NaOH aq. The solution was kept at room temperature for 1 h to remove acetyl groups from sugar residues and neutralized with 1 N AcOH aq., then concentrated. Crude product was purified by RP-HPLC to afford compound **3** (9.1 mg, 15% overall yield). MALDI-TOFMS: *m/z* calculated for $C_{58}H_{86}N_{15}O_{25}$, $[M+H]^+$ 1886.91, found 1887.25. (See also, Figure S5).

Synthesis of clusterin glycopeptide 2. An acceptor substrate, compound **3** (7.9 mg, 3.5 μmol, final; 2.5 mM), was dissolved in 200 mM (final; 100 mM) potassium phosphate

buffer (pH 6.3) and added 160 mM (final; 16 mM) SGP as donor substrate and 500 mU/mL (final; 45 mU/mL) *endo*-M. After incubation at 37°C for 1 h, the reaction mixture was subjected to the purification by RP-HPLC (retention time, 37.59 min) to give clusterin glycopeptide **2** (2.3 mg, 15%). MALDI-TOFMS: *m/z* calculated for $C_{158}H_{250}N_{26}O_{86}$, $[M\text{-H}]^-$ 3886.59, found 3886.831. (See also, Figure S4).

Synthesis of clusterin glycopeptide 1. Clusterin glycopeptide **2** (5.26 mg, 1.35 μmole) was incubated in 225.5 μL of 0.1 M HCl aq. at 80°C for 30 min according to the condition reported previously.[S7] The reaction mixture was neutralized and purified by RP-HPLC (retention time, 37.90 min) to give asialo glycopeptide **5** (2.0 mg, 45%). MALDI-TOFMS: *m/z* calculated for $C_{136}H_{216}N_{24}O_{70}$, $[M\text{-H}]^-$ 3304.4002, found 3304.802. (See also, Figure S6).

Asialo product **5** (3.40 mg, 1.03 μmole, final 1.0 mM) was dissolved in 500 mM (final; 250 nM) 4-(2-hydroxyethyl)-1-piperazineethanesulfonic acid (HEPES) buffer (pH 8.0). This solution was added to a 100 mM (final; 15 mM) CMP-Neu5Ac and 1000 U/mL (final; 75 mU/mL) recombinant α2,3-sialyltransferase, and the mixture was incubated at 37°C for 8 h. The reaction mixture was subjected directly to the purification by RP-HPLC (retention time, 37.52 min) to yield pure clusterin glycopeptide **1** (1.8 mg, 45%). MALDI-

TOFMS: *m/z* calculated for $C_{158}H_{250}N_{26}O_{86}$, [*M*-H]$^{-}$ 3886.59, found 3886.633. (See also, Figure S3).

1H NMR and HSQC spectra for the structural characterization of the synthetic glycopeptides **1**, **2**, **3**, and **5** were measured with 600 MHz and recorded at 298 K (Figure S3-S6). Based on the obtained data, the structures of all new compounds were confirmed by attributing each proton and carbon as summarized in Table S2.

Concentration of the synthetic clusterin glycopeptides. Concentrations of the standard solution of clusterin glycopeptides **1** and **2** was determined by combining amino acid analysis and RP-HPLC analysis because of the limited amounts of the glycopeptides **1** and **2** to determine the correct concentrations from their weights. Firstly, amino acid analysis of the glycopeptide **3** was carried out and the result was used for the determination of the concentration to be 590 μM (Table S3). Then, the standard solution was subjected to spike with the measurement of RP-HPLC profiles of glycopeptides **1** and **2**. By using the RP-HPLC profiles of glycopeptides **1** and **2** in the presence of concentration-defined glycopeptide **3**, the concentrations were estimated to be 168 μM and 146 μM, respectively (Figure S7 and Table S4). These solutions were employed by dilution to adjust 10 μM before the SRM experiments.

Immunoaffinity depletion of high-abundant proteins from human serum samples.[S8,S9] Before injection onto the multiple affinity removal system (MARS), human serum (15 μL) was diluted with 200 μL of Equil/Lad/Wash Buffer (Agilent Technology, Cat. No. 5185-5987). The sample was transferred to a 0.22 μm spin tube and centrifuged for 1 min at 10,000 g to remove particulates. The filtered sample was applied to Multi Affinity Removal Spin Cartridge (Agilent Technology, Cat. No. 5188-6560) and centrifuged at 100 g for 1 min. Flow-through was removed and the cartridge was kept for 5 min at room temperature. Then, Equil/Load/Wash Buffer (400 μL) was added to the cartridge and centrifuged at 100 g for 2 min (x 2 times). Flow-through fractions were collected and washed with MilliQ through an Amicon Ultra-0.5 centrifugal Filter Unit with Ultracel-50 membrane (Merck Millipore, Cat. No. UFC5050) at 12,000 g for 5 min. The residual solution was dried by centrifugal evaporator.

Reductive alkylation and tryptic digestion of the depleted serum glycoproteins. Dried sample was dissolved in 50 mM ammonium bicarbonate (100 μL) and the solution was subjected to the reduction with 1,4-dithiothreitol (DTT, 10 μL, 100 mM in MilliQ) at 60°C for 1 h, followed by alkylation with iodoacetamide (IAA, 7 μL, 123 mM in MilliQ), and incubated in dark at room temperature for 30 min. Then, the mixture was treated with sequencing grade trypsin [10 μL, trypsin/serum proteins=1/50 (w/w)] at 37°C

for 16 h.

Finally, tryptic digests were desalinated using GL-tip GC (GL Science, Cat. No. 7820-11201) as follows: The tryptic digests were applied to GL-Tip GC equilibrated with 20 μL of 5% acetonitrile in water containing 0.1% trifluoroacetic acid (TFA) and centrifuged at 3,000 g for 5 min. The column was washed with 20 μL of 5% acetonitrile in water containing 0.1% TFA and glycopeptides/peptides were eluted by centrifugation at 3,000 g for 5 min with 80 μL of 80% acetonitrile in water containing 0.1% TFA. The residual sample was dried up by centrifugal evaporator and dissolved in 15 μL of MilliQ containing 0.1% formic acid. The solution was used directly to LC-MS/MS analysis.

References

S1. Hatakeyama, S., Amano, M., Tobishima, Y., Yoneyama, T., Tsuchiya, N., Habuchi, T., Nishimura, S.-I., Ohyama, C. Serum *N*-glycan alteration associated with renal cell carcinoma detected by high throughput glycan analysis. *J. Urology* **2014**, *191*, 805-813.

S2. Premdjee, B.; Adams, A. L.; MacMillan, D. Native *N*-glycopeptide thioester synthesis through N→S acyl transfer. *Bioorg. Med. Chem. Lett.*, **2011**, *21*, 4973-4975.

S3. Hammura, K., Ishikawa, A., Ravi, K. H. V., Miyoshi, R., Yokoi, Y., Tanaka, M., Hinou, H., Nishimura, S.-I. Synthetic glycopeptides allow for the quantitation of scarce nonfucosylated IgG Fc *N*-glycans of therapeutic antibody. *ACS Med. Chem. Lett.* **2018**, *9*, 889-894.

S4. Yogesh, K. V., Kamiyama, T., Ohyama, C., Yoneyama, T., Nouso, K., Kimura, S., Hinou, H., Nishimura, S.-I. Synthetic glycopeptides as a designated standard in focused glycoproteomics to discover serum cancer biomarkers. *Med. Chem. Commun.* **2018**, *9*, 1351-1358.

S5. Ravi, K. H. V., Naruchi, K., Miyoshi, R., Hinou, H., Nishimura, S. -I. A new approach for the synthesis of hyperbranched N-glycan core structures from locust bean gum. *Org. Lett*. **2013**, *15*, 6278-6281.

S6. Matsushita, T., Hinou, H., Kurogochi, M., Shimizu, H., Nishimura, S.-I. Rapid microwave-assisted solid-phase glycopeptide synthesis. *Org. Lett.* **2005**, *7*, 877–880.

S7. Hanzawa, K.; Suzuki, N.; Natsuka, S. Structures and developmental alterations of *N*-glycans of zebrafish embryos. *Glycobiology* **2017**, *27*, 228–245.

S8. Jankovska, E., Svitek, M., Holada, K., PetrakJ. Affinity depletion versus relative protein enrichment: A side-by-side comparison of two major strategies for increasing human cerebrospinal fluid proteome coverage. *Clin. Proteomics* **2019**, *16*, 9.

S9. Zolotarjova, N., Mrozinski, P., Chen, H., Martosella, J. Combination of affinity depletion of abundant proteins and reverse-phase fractionation in proteome analysis of human plasma/serum. *J. Chromatogr. A* **2007**, *1189*, 332-338.

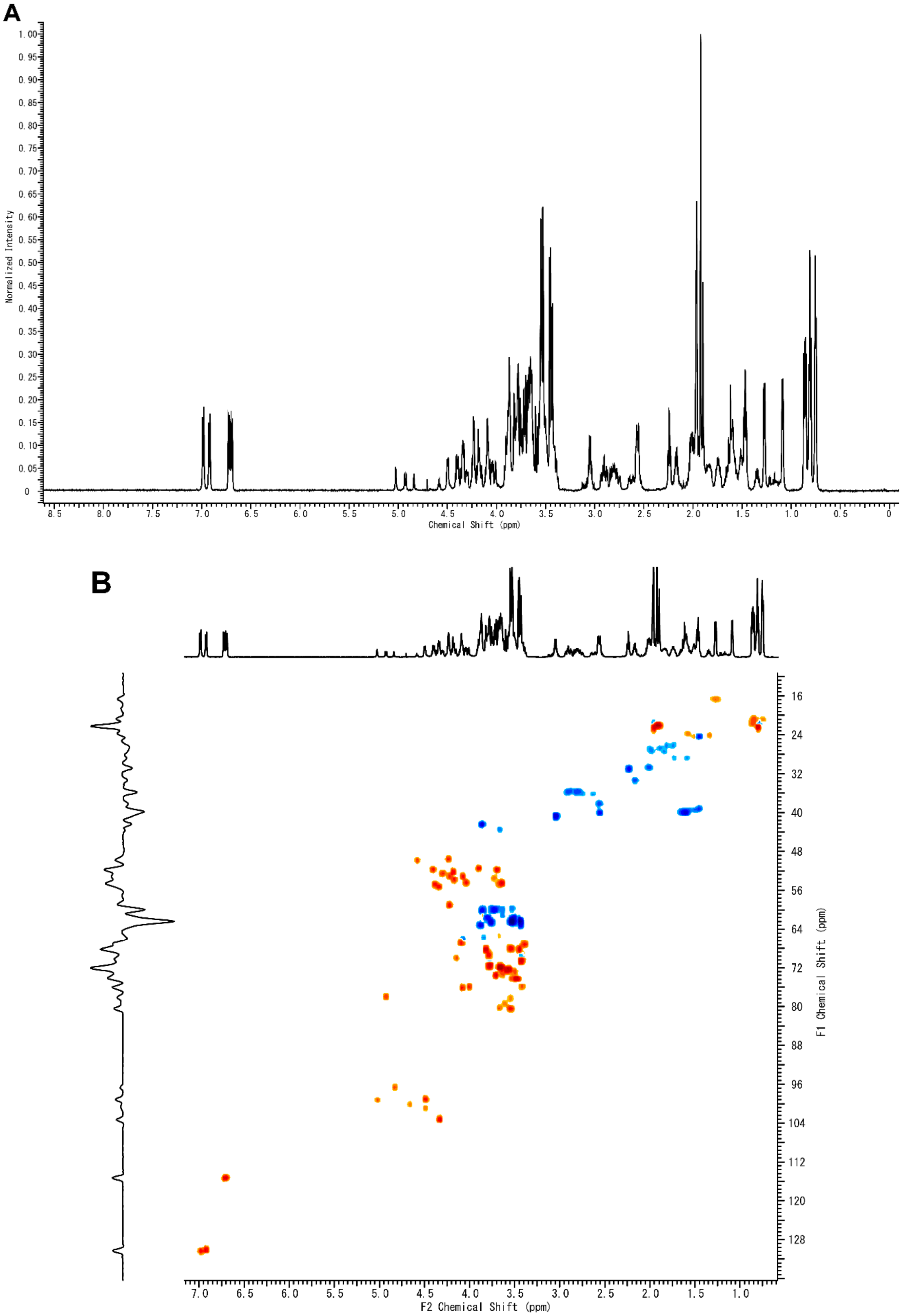

Figure S1. (A) ^{1}H-NMR and (B) HSQC (D_2O, 600 MHz, at 298 K) of compound **1**.

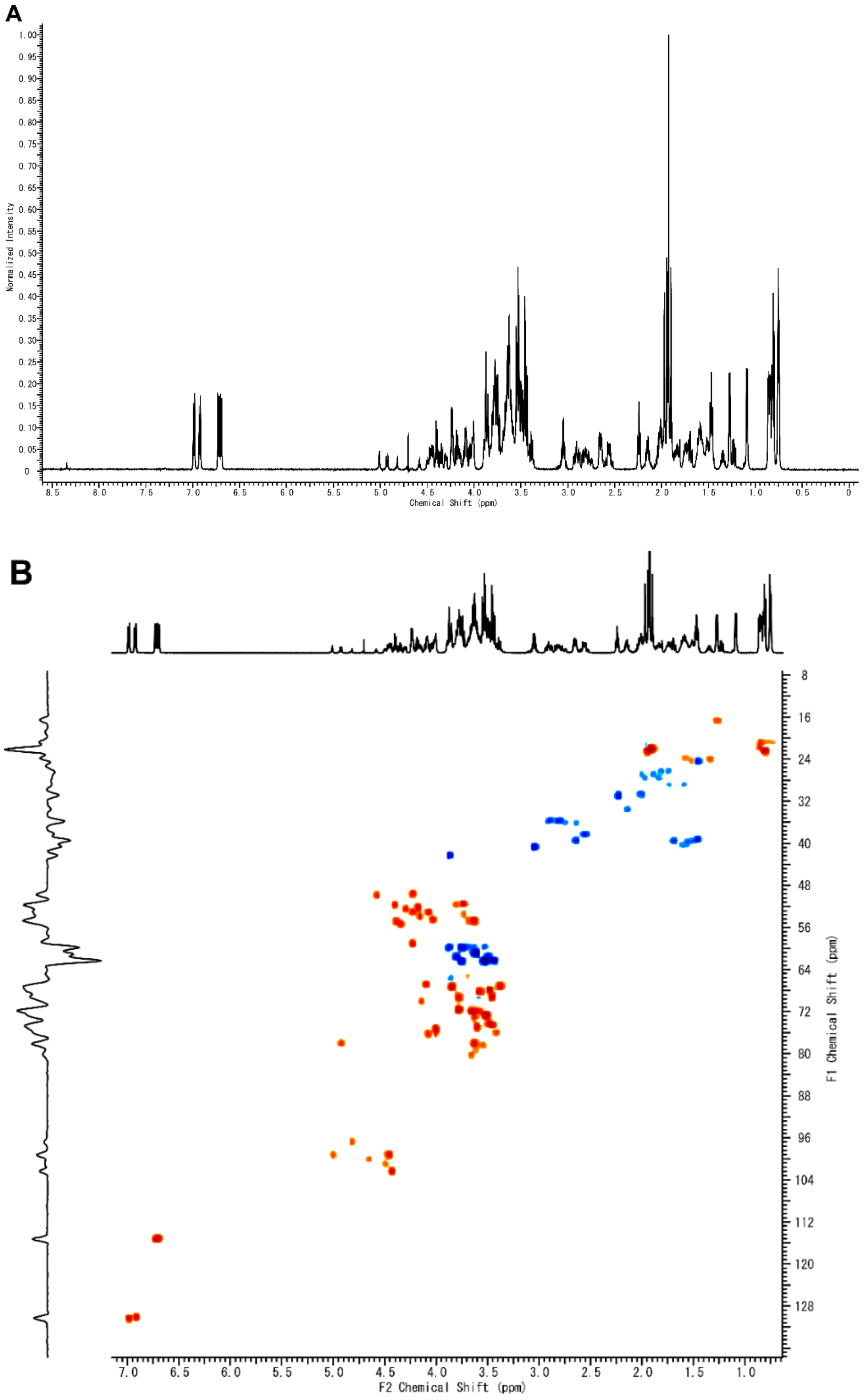

Figure S2. (A) ^{1}H-NMR and (B) HSQC (D_2O, 600 MHz, at 298 K) of compound **2**.

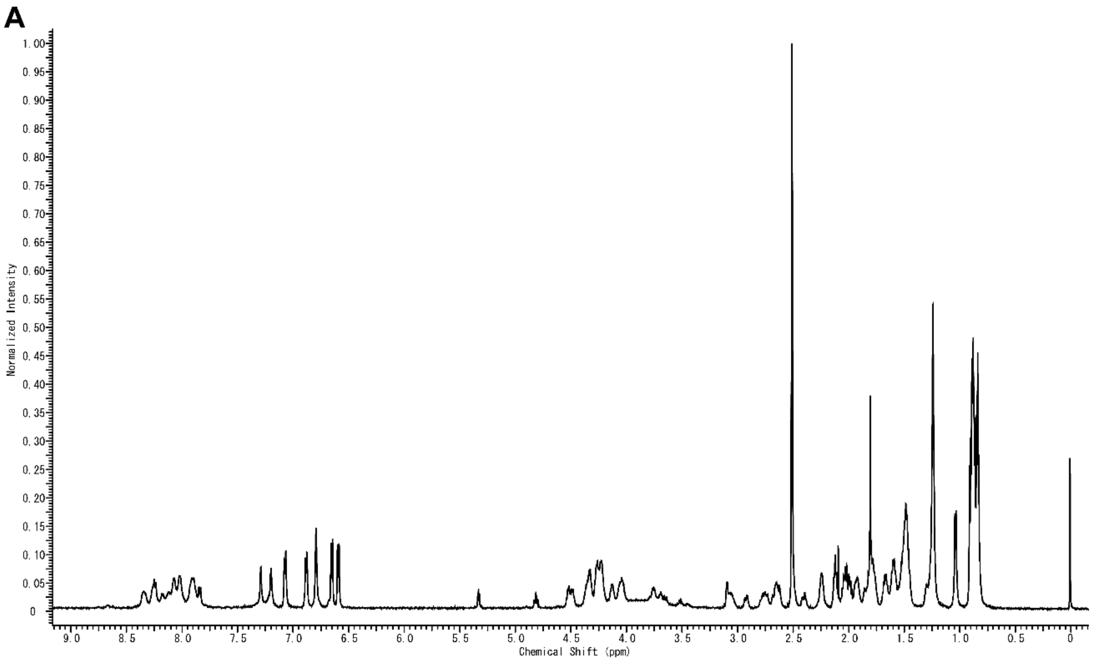

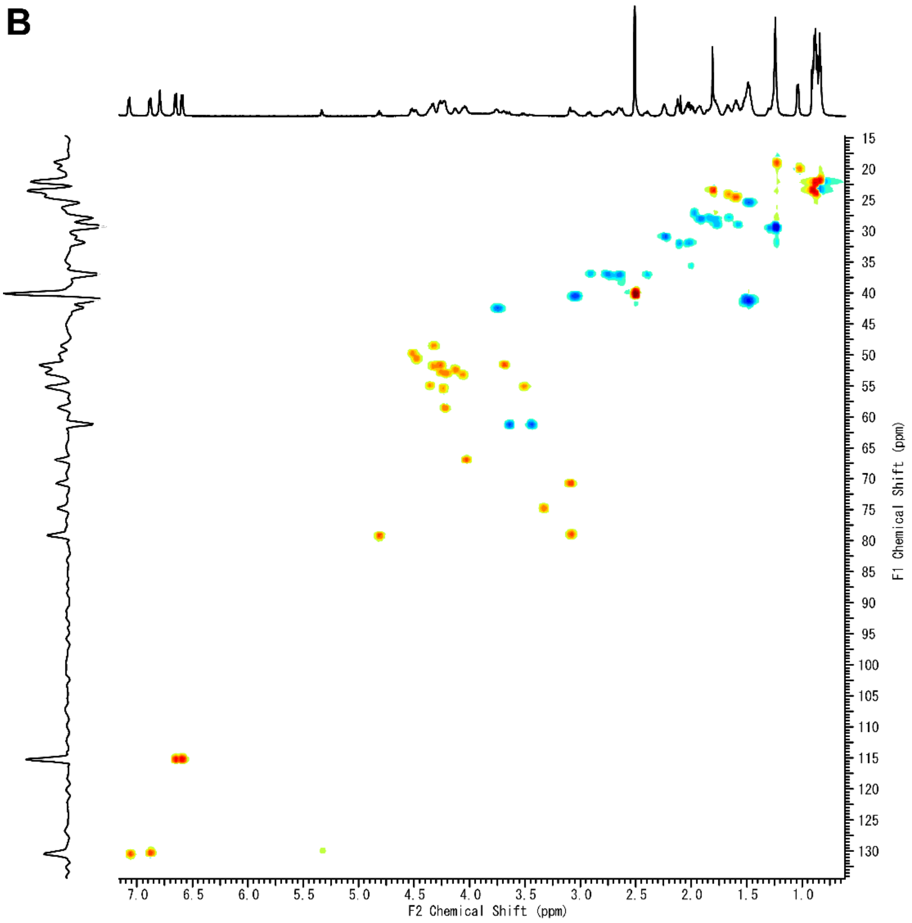

Figure S3. (A) ^{1}H-NMR and (B) HSQC (DMSO, 600 MHz, at 298 K) of compound **3**.

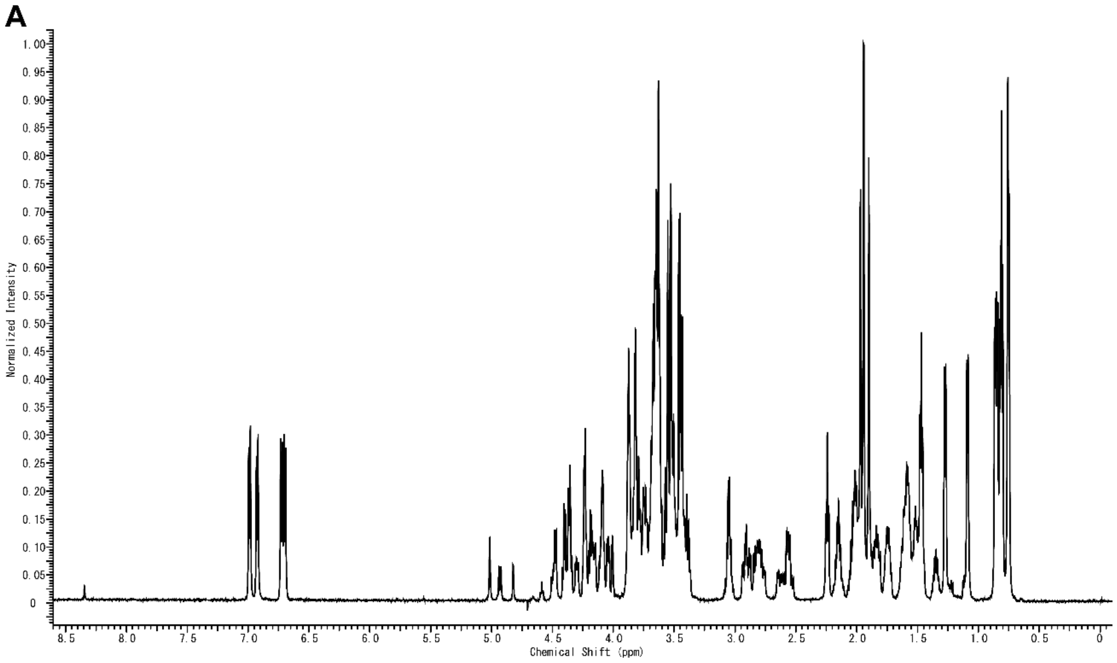

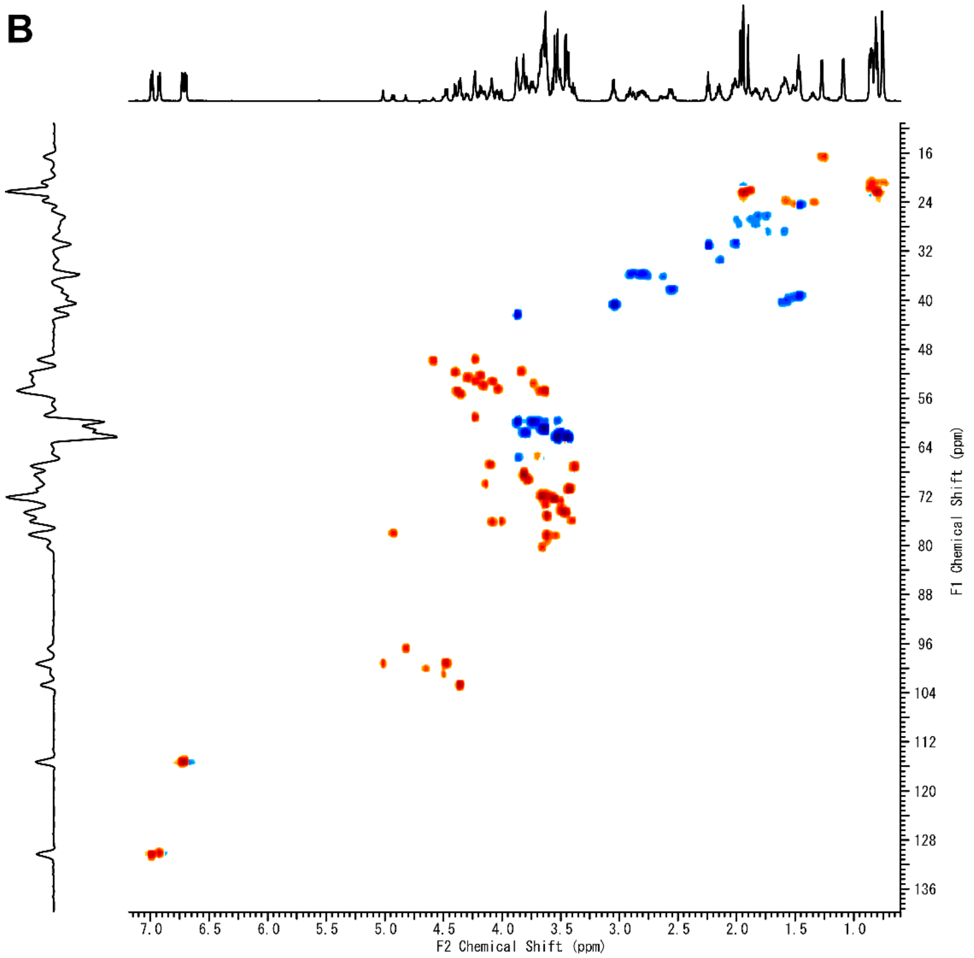

Figure S4. (A) ^{1}H-NMR and (B) HSQC (D_2O, 600 MHz, at 298 K) of compound **5**.

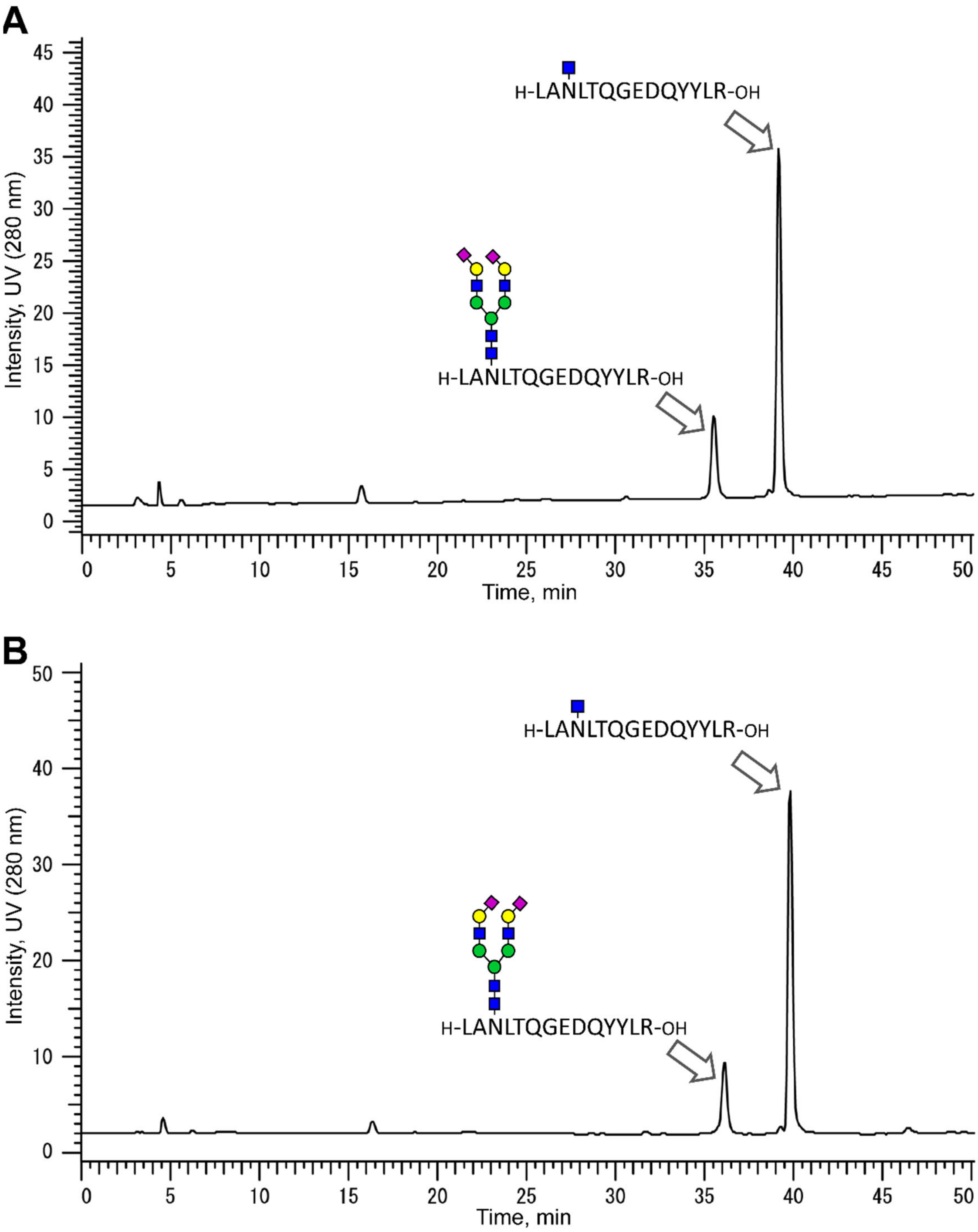

Figure S5. HPLC profile to determine the concentration of glycopeptide **1** (A) and **2** (B) under the gradient from acetonitrile/H_2O=95/5 (0 min) to acetonitrile/H_2O=70/30 (50 min).

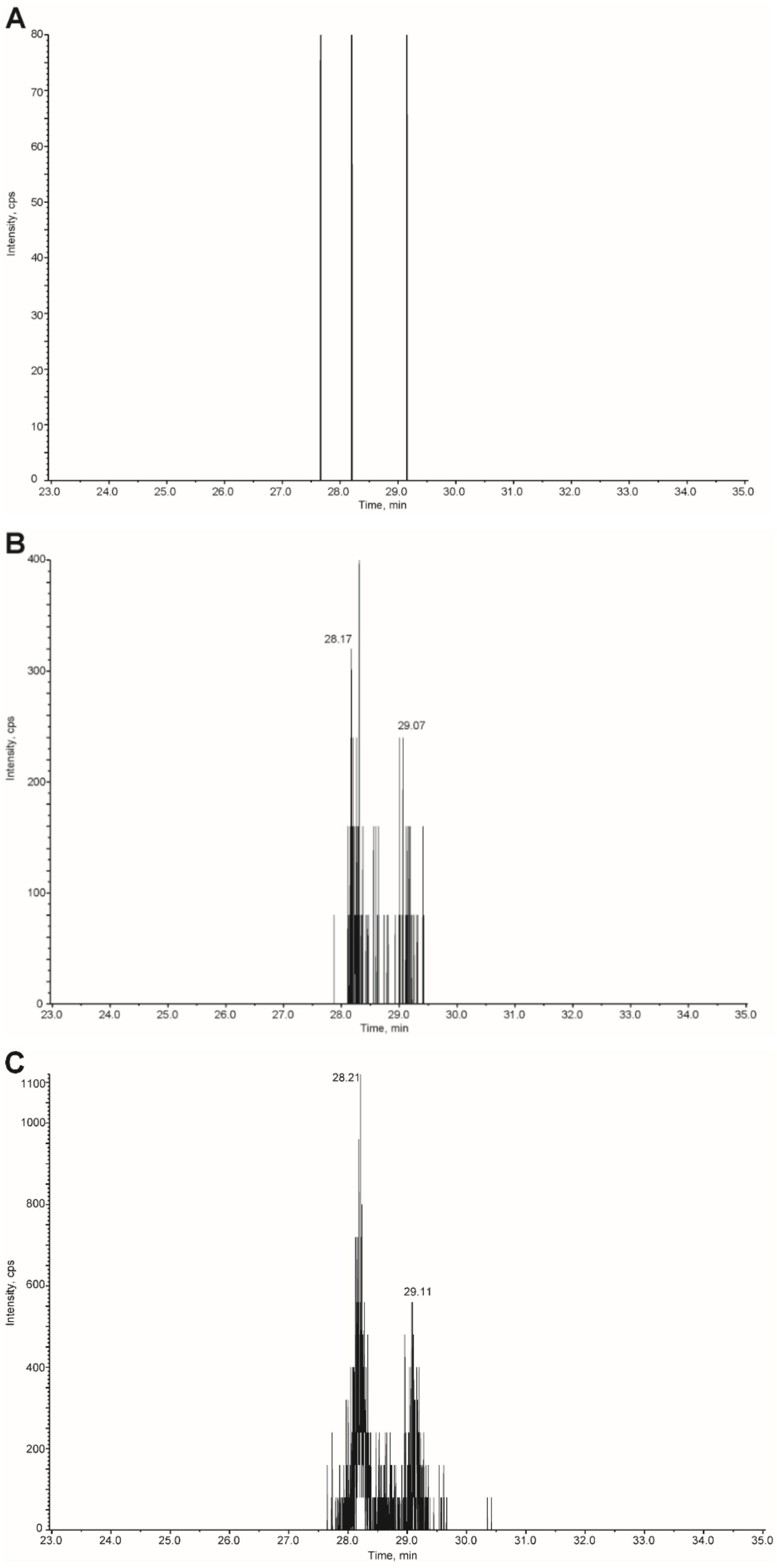
A
B
28.17
29.07
C
28.21
29.11
Intensity, cps
Time, min

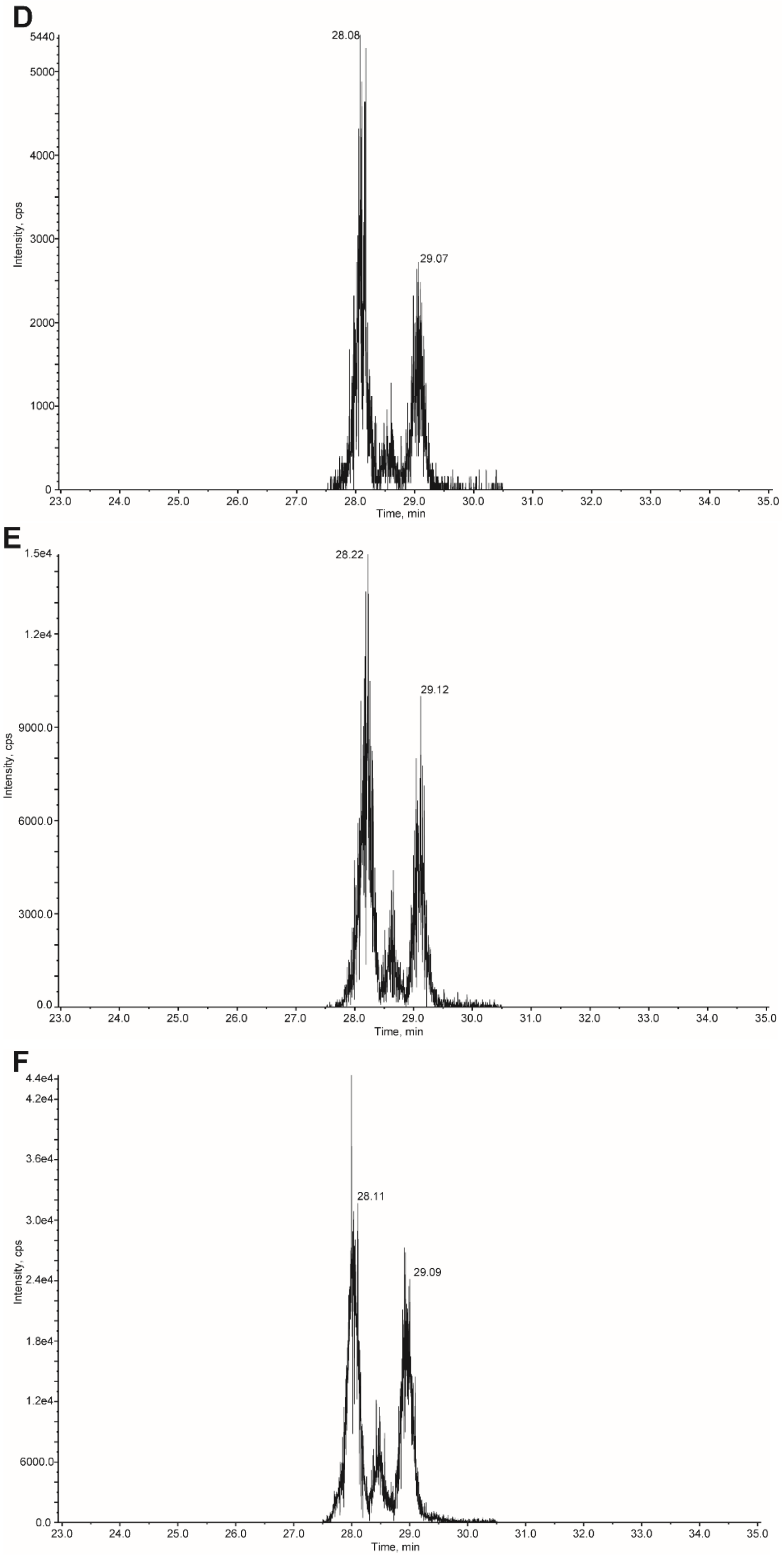

Figure S6. XICs of a series of standard mixtures based on SRM channels Q1/Q3 = 1297.2/657.4; the concentrations are 0 nM (A), 100 nM (B), 250 nM (C), 500 nM (D), 1000 nM (E) and 2500 nM (F).

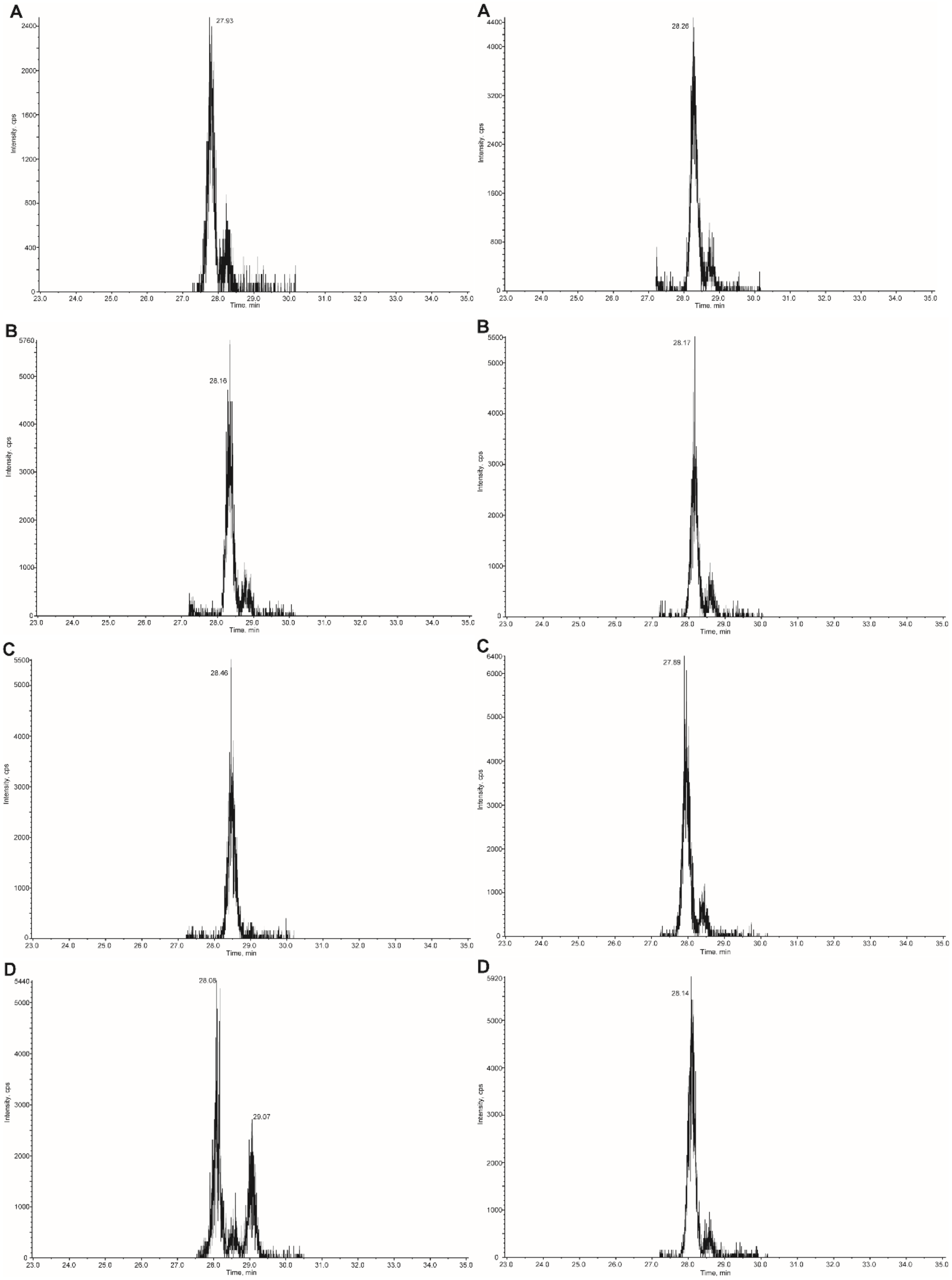
A
27.93
A
28.26
B
28.16
B
28.17
C
28.46
C
27.89
D
28.08
29.07
D
28.14
Intensity, cps
Time, min

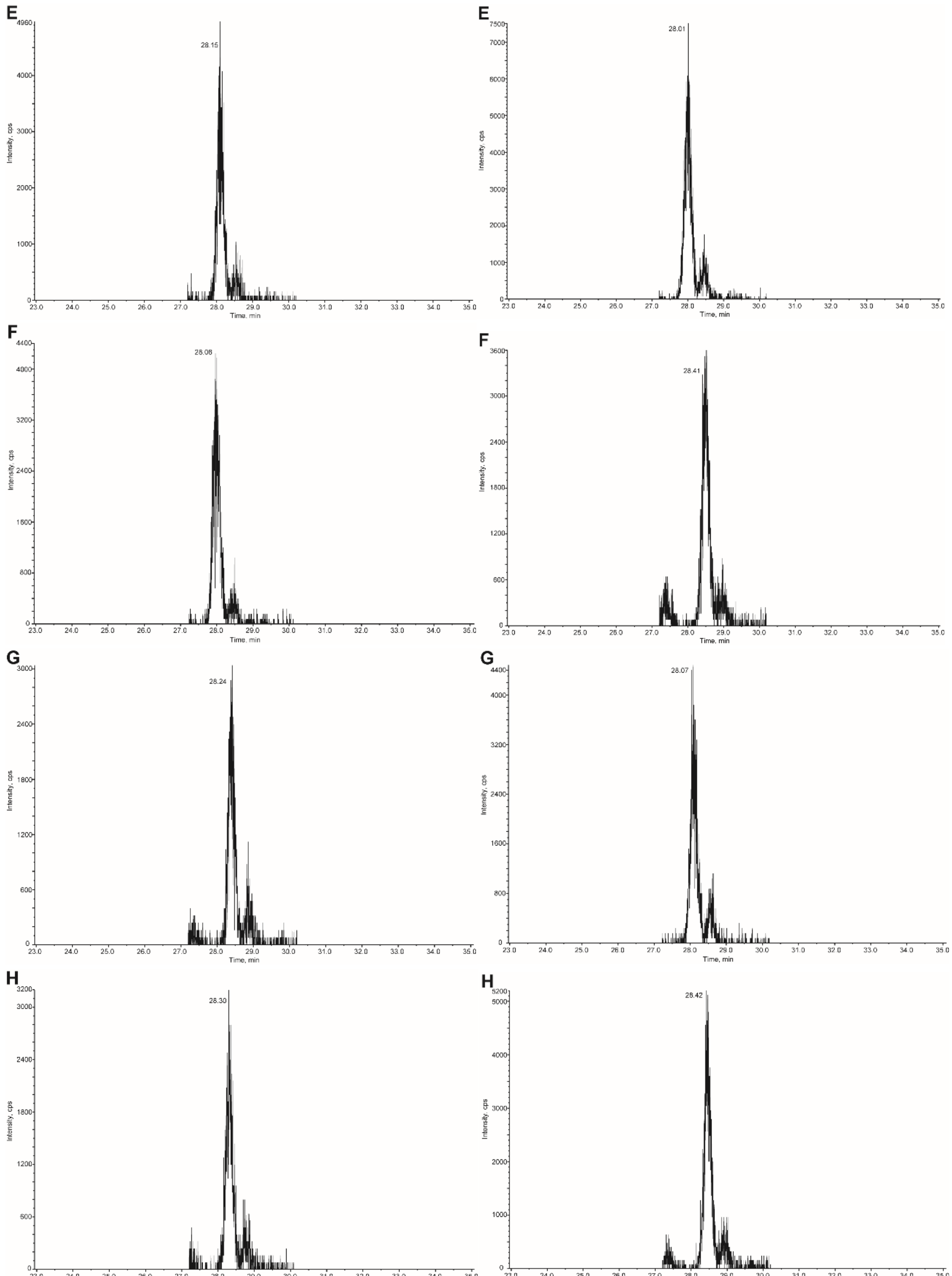

Figure S7. XICs in the SRM assay using 10 μL of serum samples from ccRCC patients (left A-H: entry 1-8) and healthy subjects (right A-H: entry 9-16) based on SRM channels Q1/Q3 = 1297.2/657.4.

Table S1. ^{1}H and ^{13}C-NMR chemical shifts of synthetic *N*-linked glycopeptides (**1**, **2**, **3**, and **5**).

Residues		**1**	**2**	**3**	**5**		**1**	**2**	**3**	**5**
Leu372	Hα	4.290	4.286	4.335	4.286	Cα	52.60	52.65	51.89	52.64
	Hβ	1.569	1.553	1.486	1.562	Cβ	39.59	39.62	41.26	40.11
	Hγ	1.507	1.504	1.597	1.503	Cγ	24.40	24.41	24.51	24.38
	Hδ	0.751	0.735	0.852	0.739	Cδ	22.42	22.44	22.22	20.87
		0.801	0.797	0.852	0.801					
	HNα	8.129	8.136	7.900	8.156					
Ala373	Hα	4.229	4.225	4.207	4.188	Cα	49.73	49.73	51.88	49.70
	Hβ	1.264	1.261	1.590	1.265	Cβ	16.64	16.71	24.53	16.69
	HNα	8.572	8.569	7.785	8.551					
Asn374	Hα	4.565	4.562	4.519	4.568	Cα	49.95	49.97	45.97	49.91
	Hβ	2.632	2.625	2.415	2.622	Cβ	36.28	36.28	37.29	36.27
		2.742	2.743	2.670	2.758					
	HNα	8.416	8.417	8.347	8.426					
	HNγ	7.044	8.525	8.242	8.533					
Leu375	Hα	4.290	4.286	4.335	4.286	Cα	52.60	52.65	51.89	52.64
	Hβ	1.569	1.553	1.486	1.562	Cβ	39.59	39.62	41.26	39.58
	Hγ	1.507	1.504	1.597	1.503	Cγ	24.40	24.41	24.51	23.92
	Hδ	0.751	0.735	0.852	0.739	Cδ	22.42	22.44	22.22	20.83
		0.801	0.797	0.852	0.801					
	HNα	8.129	8.136	7.900	8.156					
Thr376	Hα	4.219	4.215	4.228	4.218	Cα	59.16	59.18	58.66	59.18
	Hβ	4.077	4.085	4.038	4.089	Cβ	66.93	66.95	67.06	66.92
	Hγ	1.081	1.078	1.038	1.083	Cγ	18.93	18.81	20.16	18.88
	HNα	8.123	8.117	7.882	8.115					
Gln377	Hα	4.221	4.215	4.267	4.221	Cα	53.23	53.24	52.81	53.22
	Hβ	1.883	1.882	1.778	1.881	Cβ	26.87	26.86	28.05	31.08
		1.997	1.993	1.922	1.997					
	Hγ	2.233	2.231	2.245	2.233	Cγ	31.29	31.10	31.11	26.85
	HNα	8.294	8.285	8.174	8.284					
	HNδ	-	-	6.873	-					
Gly378	Hα	3.861	3.857	3.757	3.863	Cα	42.45	42.49	42.53	42.45
	HNα	8.329	8.328	8.263	8.321					

Residues		1	2	3	5		1	2	3	5
Glu379	Hα	4.161	4.164	4.219	4.157	Cα	54.03	53.89	53.05	54.03
	Hβ	1.841	1.837	1.800	1.826	Cβ	27.73	27.57	28.09	33.56
		1.979	1.977	1.930	1.965					
	Hγ	2.152	2.162	2.121	2.154	Cγ	33.62	33.46	31.77	27.76
	HNα		8.262	8.071	8.111					
Asp380	Hα	4.393	4.393	4.484	4.393	Cα	51.86	51.81	50.71	51.85
	Hβ	2.550	2.552	2.502	2.547	Cβ	38.35	38.30	38.46	38.86
		2.550	2.552	2.642	2.547					
	HNα	8.346	8.344	8.331	8.343					
Gln381	Hα	4.075	4.074	4.062	4.072	Cα	53.26	53.29	53.21	53.25
	Hβ	1.738	1.738	1.668	1.739	Cβ	26.31	26.35	27.90	30.75
		1.803	1.805	1.838	1.800					
	Hγ	2.012	2.010	2.041	2.014	Cγ	30.77	30.77	31.93	26.33
	HNα	8.116	8.113	8.120	8.223					
	HNδ	-	-	6.615	-					
Tyr382	Hα	4.336	4.333	4.243	4.337	Cα	55.44	55.39	55.53	55.45
	Hβ	2.782	2.779	2.648	2.784	Cβ	35.84	35.74	37.21	35.69
		2.870	2.867	2.744	2.871					
	H(2,6)	6.903	6.900	6.883	6.914	C(2,6)	130.3	130.3	130.5	130.3
	H(3,5)	6.686	6.682	6.594	6.707	C(3,5)	115.4	115.4	115.3	115.4
	HNα	8.003	7.999	8.019	8.004					
Tyr383	Hα	4.337	4.375	4.360	4.380	Cα	54.94	54.94	55.07	54.91
	Hβ	2.814	2.811	2.774	2.815	Cβ	35.93	35.93	37.05	35.87
		2.907	2.905	2.922	2.908					
	H(2,6)	6.976	6.971	7.069	6.977	C(2,6)	130.5	130.5	130.7	130.5
	H(3,5)	6.713	6.714	6.652	6.747	C(3,5)	115.5	115.5	115.4	115.5
	HNα	7.645	7.647	8.069	7.646					
Leu384	Hα	4.173	4.171	4.264	4.175	Cα	52.34	52.35	51.79	52.35
	Hβ	1.457	1.454	1.511	1.458	Cβ	39.37	39.33	41.14	39.36
	Hγ	1,340	1.338	1.584	1.341	Cγ	24.12	24.16	24.51	24.14
	Hδ	0.739	0.735	0.879	0.741	Cδ	20.80	20.82	22.31	22.43
		0.789	0.785	0.879	0.789					
	HNα	7.842	7.842	8.017	7.841					

Residues		**1**	**2**	**3**	**5**		**1**	**2**	**3**	**5**
Arg385	Hα	4.030	4.028	4.130	4.030	Cα	54.60	54.58	52.46	54.61
	Hβ	1.590	1.587	1.578	1.587	Cβ	28.90	28.93	28.98	28.92
		1.726	1.723	1.773	1.728					
	Hγ	1.456	1.454	1.492	1.453	Cγ	24.51	24.50	25.80	24.52
	Hδ	3.035	3.036	3.064	3.038	Cδ	40.76	40.77	40.70	40.77
	HNα	7.653	7.652	7.925	7.650					
	HNδ	7.044	7.041	8.247	7.041					
GlcNAc1	H1	4.926	4.927	4.815	4.933	C1	78.16	78.16	79.35	78.12
	H2	3.739	3.734	3.518	3.736	C2	53.64	53.66	55.24	53.64
	H3	3.634	3.637	3.337	3.569	C3	73.18	72.94	74.88	72.70
	H4	3.553	3.548	3.099	3.626	C4	78.49	78.53	70.84	78.38
	H5	3.421	3.419	3.451	3.415	C5	76.11	76.03	74.50	76.11
	H6	3.535	3.532	3.448	3.652	C6	59.78	59.83	61.27	61.01
		3.712	3.698	3.650	3.721					
	HN	8.100	8.103	7.838	8.097					
GlcNAc2	H1	4.497	4.496		4.502	C1	101.1	101.1		101.1
	H2	3.656	3.648		3.670	C2	54.87	54.68		54.90
	H3	3.663	3.661		3.631	C3	72.00	72.01		72.04
	H4	3.625	3.617		3.627	C4	79.35	79.61		79.34
	H5	3.493	3.487		3.496	C5	74.41	74.36		74.38
	H6	3.757	3.733		3.652	C6	59.93	60.23		59.83
		3.881	3.867		3.721					
	HN	8.334	8.330		8.340					
Man3	H1	4.655	4.665		4.658	C1	100.3	100.3		100.3
	H2	4.142	4.150		4.146	C2	70.11	70.11		70.1
	H3	3.667	3.672		3.667	C3	80.36	80.48		80.39
	H4	3.506	3.530		3.511	C4	74.33	74.24		72.81
	H5	3.697	3.673		3.705	C5	65.41	65.49		65.51
	H6	3.696	3.670		3.659	C6	65.74	65.86		65.75
		3.856	3.850		3.859					

Residues		1	2	3	5		1	2	3	5
Man4	H1	5.009	5.025		5.016	C1	99.40	99.38		99.38
	H2	4.085	4.090		4.088	C2	76.37	76.31		76.32
	H3	3.787	3.789		3.799	C3	69.41	69.46		69.16
	H4	3.386	3.389		3.401	C4	67.28	67.33		67.15
	H5	3.522	3.509		3.639	C5	72.82	72.82		73.39
	H6	3.504	3.513		3.508	C6	61.72	61.67		61.65
		3.806	3.803		3.809					
Man4'	H1	4.819	4.839		4.824	C1	96.94	96.77		96.87
	H2	4.011	4.009		4.007	C2	75.55	76.12		76.23
	H3	3.787	3.789		3.789	C3	69.41	69.46		69.39
	H4	3.386	3.399		3.388	C4	67.28	67.36		67.30
	H5	3.522	3.509		3.510	C5	72.82	72.82		72.86
	H6	3.504	3.513		3.508	C6	61.72	61.67		61.65
		3.806	3.803		3.809					
GlcNAc5	H1	4.467	4.495		4.477	C1	99.36	99.20		99.33
	H2	3.642	3.689		3.643	C2	54.84	54.73		54.86
	H3	3.663	3.661		3.667	C3	72.00	72.01		71.99
	H4	3.638	3.548		3.554	C4	78.19	80.56		78.48
	H5	3.455	3.487		3.473	C5	74.71	74.36		74.58
	H6	3.757	3.740		3.743	C6	59.93	60.02		59.94
		3.881	3.859		3.873					
	HN	8.109	8.120		8.110					
GlcNAc5'	H1	4.467	4.495		4.477	C1	99.36	99.20		99.33
	H2	3.642	3.689		3.643	C2	54.84	54.73		54.86
	H3	3.663	3.661		3.667	C3	72.00	72.01		71.99
	H4	3.638	3.548		3.554	C4	78.19	80.56		78.48
	H5	3.455	3.487		3.473	C5	74.71	74.36		74.58
	H6	3.757	3.740		3.743	C6	59.93	60.02		59.94
		3.881	3.859		3.873					
	HN	8.157	8.120		8.156					

Residues		1	2	3	5		1	2	3	5
Gal6	H1	4.441	4.336		4.634	C1	102.5	103.4		102.8
	H2	3.463	3.427		3.453	C2	69.33	70.66		70.94
	H3	3.632	3.560		3.565	C3	71.99	72.23		72.42
	H4	3.850	3.818		3.820	C4	67.40	68.34		68.51
	H5	3.606	3.714		3.623	C5	75.10	73.62		75.24
	H6	3.448	3.434		3.449	C6	62.40	63.28		62.41
		3.773	3.884		3.535					
Gal6'	H1	4.441	4.336		4.634	C1	102.5	103.4		102.8
	H2	3.463	3.427		3.453	C2	69.33	70.66		70.94
	H3	3.632	3.560		3.565	C3	71.99	72.23		72.42
	H4	3.850	3.818		3.820	C4	67.40	68.34		68.51
	H5	3.606	3.714		3.623	C5	75.10	73.62		75.24
	H6	3.448	3.434		3.449	C6	62.40	63.28		62.41
		3.773	3.884		3.535					
Neu5Ac7	H3(ax)	1.697	1.599			C3	39.57	40.09		
	H3(eq)	2.653	2.552							
	H4	3.579	3.546			C4	68.39	68.18		
	H5	3.743	3.700			C5	51.68	51.86		
	H6	3.586	3.585			C6	72.04	72.45		
	H7	3.488	3.453			C7	68.03	68.32		
	H8	3.788	3.783			C8	71.74	71.62		
	H9	3.534	3.533			C9	62.47	62.62		
		3.767	3.767							
	HN	7.955	7.933							
Neu5Ac7'	H3(ax)	1.697	1.599			C3	39.57	40.09		
	H3(eq)	2.653	2.552							
	H4	3.579	3.546			C4	68.39	68.18		
	H5	3.743	3.700			C5	51.68	51.86		
	H6	3.586	3.585			C6	72.04	72.45		
	H7	3.488	3.453			C7	68.03	68.32		
	H8	3.788	3.783			C8	71.74	71.62		
	H9	3.534	3.533			C9	62.47	62.62		
		3.767	3.767							
	HN	7.972	7.933							

Table S2. Recommended default mass parameters used in this study

	TurboIonSpray	
	5-10 μL/min	200 μL/min
Curtain Gas	10	40
Collision Gas	High	High
IonSpray Voltage (Positive)	5000	5500
Temperature	0	400
Ion Source Gas1	20	50
Ion Source Gas2	0	80
Interface Heater	on	off
Declustering Potential	70	70
Entrance Potential	10	10

Table S3. The result of amino acid analysis of glycopeptide **3**

amino acid	concentration (nmol/40 μL)
Asp	11.94631
Thr	5.69843
Ser	0.03384
Glu	17.87096
Gly	5.93202
Ala	5.85351
Cystine	0.01410
Val	0.01973
Leu	17.66651
Nle	1.08671
Tyr	11.80230
Phe	0.01977
Lys	0.01866
His	0.01369
NH_3	21.35955
Arg	5.98819

Table S4. Peak areas and calculated concentrations from HPLC spectrums in Figure **S7**

glycopeptide	peak area	concentration (μM)
**3**	550182	590
1	147108	168
**3**	514549	590
**2**	136567	146

Table S5. Information of serum samples and the results of SRM assay

Entry	Clinical diagnosis	Sex	Age	TNM classification	Conc. of clusterin with glycopeptide **2** [nM]
1	RCC	male	60's	T1aN0M1	315.3
2	RCC	female	70's	T1bN1M0	513.3
3	RCC	male	70's	T3N0M0	461.8
4	RCC	male	40's	T1aN0M0	461.1
5	RCC	male	40's	T1aN0M0	488.2
6	RCC	male	40's	T1aN0M0	538.9
7	RCC	female	60's	T1aN0M0	354.9
8	RCC	male	60's	T3bN0M1	363.7
9	healthy	male	50's	—	465.9
10	healthy	male	50's	—	440.9
11	healthy	male	50's	—	574.0
12	healthy	male	60's	—	601.8
13	healthy	female	50's	—	697.5
14	healthy	male	60's	—	458.1
15	healthy	male	50's	—	503.5
16	healthy	female	60's	—	561.4

Serum samples from 8 RCC patients (entry 1-8) were collected before treatment at Hirosaki University and 8 healthy subjects (entry 9-16) were collected at Showa University Hospital, respectively. Immediately after serum collection, samples were aliquoted, frozen, and stored at -80°C until pre-processing by an experimental workflow.
